# CD44 and HAP‐Conjugated hADSCs as Living Materials for Targeted Tumor Therapy and Bone Regeneration

**DOI:** 10.1002/advs.202206393

**Published:** 2023-05-08

**Authors:** He Xia, Min Hao, Kaiwen Li, Xin Chen, Liyang Yu, Jichuan Qiu, Hongyu Zhang, Haijun Li, Yuanhua Sang, Hong Liu

**Affiliations:** ^1^ State Key Laboratory of Crystal Materials Shandong University Jinan 250100 P. R. China; ^2^ Department of Geriatrics and the Key Laboratory of Magnetic Field‐free Medicine and Functional Imaging (MF) Qilu Hospital Cheeloo College of Medicine Shandong University Jinan 250100 P. R. China

**Keywords:** bone tissue regeneration, living material, stem cells, targeted tumor therapy

## Abstract

Combining targeted tumor therapy with tissue regeneration represents a promising strategy for synergistic tumor therapy. In this study, a multifunctional living material is constructed with human‐derived adipose stem cells (hADSCs) and antibody‐modified hydroxyapatite nanorods (nHAP) for targeted drug delivery and bone regeneration following surgery. The living material delivers the therapeutics to the tumor site efficiently based on the strength of the inherent tumor tropism of hADSCs. The bioconjugation of nHAP with hADSCs via specific antibody modification is found to be biocompatible, even when loaded with the chemotherapeutic drug doxorubicin (Dox). The endocytosis of nHAP stimulates the osteogenic differentiation of hADSCs, promoting bone tissue regeneration. Moreover, the antibody‐modified nHAP‐hADSC conjugate exhibits targeted tumor delivery, which is further facilitated by pH‐triggered release of Dox, inducing apoptosis of tumor cells with low toxicity to healthy tissues. Therefore, the present study provides a general strategy for engineering living materials to achieve targeted tumor therapy and bone tissue regeneration after surgery, which can be extended to other disease types.

## Graphical abstract

Here, engineered living material is constructed by living cells (human adipose–derived stem cells, hADSCs) and biomaterials (doxorubicin‐loaded Anti‐CD44 antibody‐modified hydroxyapatite) with dual functions of targeted residual tumor elimination and bone regeneration after surgery. The living material provides a general strategy for next‐generation targeted tumor therapy and bone tissue regeneration.

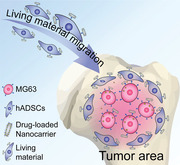

## 1 Introduction

Osteosarcoma is the most prevalent primary malignant bone tumor and predominantly observed in adolescents. Approximately half of the patients have metastatic disease, which limits the efficacy of available treatments. Despite ongoing efforts, therapeutic progress for osteosarcoma has been largely stagnant since the 1980s.^[^ ^1^ ^]^ Currently, surgical resection of the primary tumor and chemotherapy are the primary modalities used for management.^[^ ^2^ ^]^ Inevitably, residual tumor cells after surgical resection pose a great risk of relapse.^[^ ^3^ ^]^ The survival of patients with inoperable relapsed and/or metastatic osteosarcoma is dismal.^[^ ^4^ ^]^ Although chemotherapy has been effective in eradicating inoperable and metastatic tumors, the lack of selectivity of chemotherapeutic agents can cause damage to healthy tissues and negatively affect bone reconstruction. Therefore, it is of great significance to suppress tumor relapse by reconstructing the affected bone. By occupying the lesions, the microenvironment is adjusted, and the scattered tumor cells are further inhibited. Therefore, targeted drug delivery to the tumor and efficient bone regeneration are both urgently needed for effective osteosarcoma therapy. Thus, it is imperative to explore novel approaches to achieve synergistic tumor therapy.

Conventional targeted therapy depends on molecular targeted drugs, but its clinical application is limited by on‐target toxicity and off‐target toxicity. On‐target toxicity refers to the side effect of the targeted drug, which lacks of selectivity between cancerous and healthy tissues. For example, kinase inhibitors affect all cells with molecular targets, including those in healthy tissues. Moreover, molecular targeted drugs also encounter off‐target problems.^[^ ^5^ ^]^ Hence, it is crucial to develop an optimal drug delivery vehicle that minimizes the drug dose, improves the selectivity of tumor sites, and reduces off‐target toxicity. With the integration of material science, various nanoparticles have been developed as drug vehicles to enable active targeting of the tumor site based on the enhanced permeability and retention (EPR) effect.^[^ ^6^ ^]^ However, nanoparticle‐based delivery systems are only effective in well‐vascularized tumors and are ineffective for metastatic tumors.^[^ ^7^ ^]^ Additionally, some studies have suggested that only 0.7% of injected nanoparticles systemically reach the tumor microenvironment, which is not as efficient as the classical EPR effect.^[^ ^8^ ^]^ Therefore, a strategy with active drug vehicles is urgently needed to enhance drug delivery efficiency.

The latest generation of therapeutics considers live cells as active vehicles for nanoformulation.^[^ ^9^ ^]^ As reported, living bacteria,^[^ ^10^ ^]^ macrophages,^[^ ^11^ ^]^ and neutrophils^[^ ^12^ ^]^ have been engineered to deliver therapeutic payloads in tumor therapy. However, most living cells load nanoformulations by internalization, which can negatively impact their viability and function. To address this limitation, a specific loading strategy of nanoformulation onto living cells could be tailored using biochemical reactions.^[^ ^13^ ^]^ The concept of living material has been proposed, which involves combining live cells and functional materials through specific antigen–antibody biochemical reactions.^[^ ^14^ ^]^ In our previous work, we modified mesoporous silica‐coated superparamagnetic Fe_3_O_4_ nanoparticles with Anti‐CD44 antibodies to tightly bind to human adipose–derived stem cells (hADSCs), resulting in living materials with high drug delivery efficiency.^[^ ^15^ ^]^ Notably, as discussed above, the occupancy of lesions as soon as possible is critical for efficient osteosarcoma therapy. Therefore, spontaneous bone regeneration would play an indispensable role in enhancing tumor treatment after surgery.

Stem cells possess a remarkable ability to self‐renew and differentiate into specific cell types, making them attractive for accelerating tissue regeneration. Combined with their natural tumor tropic properties, stem cells are one of the best candidates to serve as tumor‐targeted vehicles and lesion occupancy agents.^[^ ^16^ ^]^ hADSCs are easily isolated from adipose tissue and exhibit great immunomodulatory properties and natural tumor‐tropic migratory properties in response to tumor‐secreted chemoattractants, angiogenic factors, and/or inflammatory signals.^[^ ^17^, ^18^, ^19^, ^20^ ^]^ As such, loading nanoformulations onto hADSCs can improve the accessibility of antitumor agents to tumors and/or their metastases, presenting a potential avenue for tumor therapy and tissue regeneration.

The tailored design of nanoformulations composed of nanocarriers and drugs is also crucial for multifunctional targeted therapy and tissue regeneration. Hydroxyapatite (Ca_10_(PO_4_)_6_(OH)_2_, HAP) nanoparticles, homologous materials in bone, can be precisely formulated and identified as preferable drug carriers due to their prominent drug‐loading capacity and great biocompatibility.^[^ ^21^ ^]^ Furthermore, HAP nanoparticles have been shown to inhibit tumor cell metabolism and induce apoptosis in various tumor cells, including osteosarcoma cells, breast cancer cells, and melanoma cells.^[^ ^22^ ^]^ Moreover, HAP nanoparticles have been widely confirmed as inorganic factors that stimulate osteogenic differentiation.^[^ ^23^ ^]^ Thus, HAP nanoparticles can serve as both nanocarriers for delivering therapeutic drugs and therapeutic agents to promote tumor therapy and tissue regeneration.

**Scheme 1:**
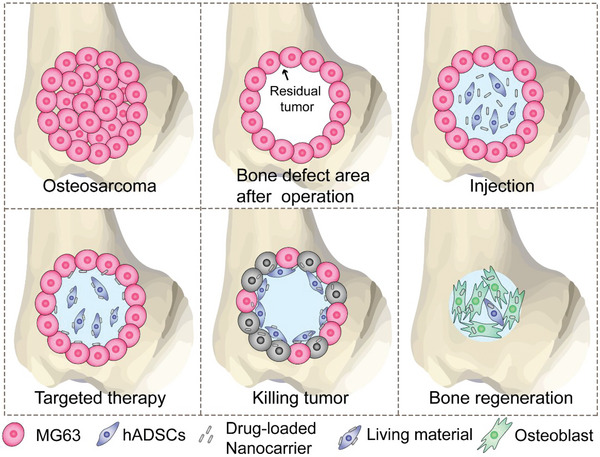
Design of the living material for the proposed targeted residual tumor elimination and bone regeneration after surgery.

In the present study, an engineered living material, composed of stem cells, antibody‐modified nanocarriers, and drugs, was proposed with dual functions of targeted residual tumor elimination and bone regeneration after surgery (**Scheme** **1**). Briefly, HAP nanorods were synthesized as nanocarriers for doxorubicin (Dox), a chemical drug, loaded by physical absorption. Anti‐CD44 monoclonal antibodies were conjugated with HAP nanorods by covalent bonding using 1,2‐dichloroethane (EDC)/NHS chemistry. First, the antibody‐modified HAP nanorods were conjugated to hADSCs based on specific antigen–antibody biochemical reactions. The living material constructed with stem cells provided an active driving force to deliver the Dox‐loaded HAP nanorods to the tumor area. In addition, the antibody‐modified nanocarrier functioned as a targeted antitumor agent that synergized with Dox to suppress tumor growth. Moreover, the internalized HAP nanorods also promoted osteogenic differentiation of hADSCs, thus facilitating bone regeneration. In summary, the antibody‐modified living material exhibited excellent antitumor and osteogenic capabilities, representing a promising strategy for next‐generation targeted tumor therapy and bone tissue regeneration.

## 2 Results and Discussion

### 2.1 Anti‐CD44 Antibody‐Modified HAP Nanorods and Dox Loading

**Figure 1:**
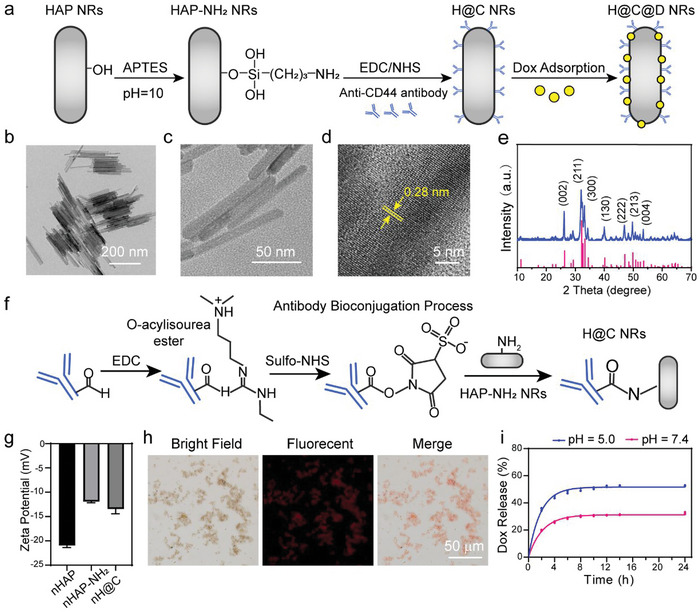
Characteristics of Dox‐loaded Anti‐CD44 antibody‐modified nHAP. a) Scheme of the preparation process of Dox‐loaded Anti‐CD44 antibody‐modified nHAP. b,c) Transmission electron microscopy (TEM) images of nHAP. d) High‐resolution transmission electron microscopy (HRTEM) images of nHAP. e) XRD pattern of nHAP. f) Scheme of the bioconjugation process between the antibody and nHAP‐NH_2_. g) Zeta potentials of nHAP, nHAP–NH_2_, and nHAP@Anti‐CD44 antibody (*n* = 3). h) Images of rhodamine B‐labeled H@C captured by an inverted fluorescence microscope. i) Dox release from H@C@D in pH 7.4 and pH 5.0 solutions.

Hydroxyapatite nanorods (nHAP) were synthesized by a hydrothermal method as previously described and were further modified with a human Anti‐CD44 antibody. Subsequently, Dox was loaded on nHAP by physical adsorption to obtain Dox‐loaded Anti‐CD44 antibody‐modified nHAP (H@C@D) as illustrated in **Figure** **1**a. Transmission electron microscopy (TEM) images (Figure 1b,c) of the synthesized nHAP showed uniform rod‐shaped nanoparticles with a high aspect ratio. The diameter was ≈10 nm, and the length was ≈100–150 nm. The high‐resolution transmission electron microscope (HRTEM) image confirmed the highly crystalline structure of the obtained nHAP (Figure 1d). The scanning electron microscopy (SEM) images showed that the antibody modification did not affect the morphology of HAP nanorods (Figure S1a, Supporting Information). The X‐ray diffraction (XRD) peaks of the synthesized products were assigned to the standard nHAP phase (ICSD card no. 74–0566, Figure 1e). Biochemical modification with human Anti‐CD44 antibody was achieved by the specific EDC/sulfo‐NHS method (Figure 1f). The zeta potential of the nanorods changed from −21.0 to −11.9 mV, indicating the successful amine modification of nHAP. By antibody conjugation, the zeta potential also slightly changed (Figure 1g). Moreover, the rhodamine B–labeled Anti‐CD44 antibody‐modified nHAP presented strong red fluorescence, which further indicated the achievement of antibody modification (Figure 1h). Successful Dox loading was confirmed by ultraviolet–visible (UV–vis) spectroscopy at 482 nm, and the standard curve of Dox release was obtained (Figure S2, Supporting Information). As shown in Figure 1i, the percentages of Dox released at pH 7.4 and 5.0 were 19.7% and 35.4% in the initial 2 h, respectively. Over a period of 8 h, Dox release reached 30.1% at pH 7.4 and 50.0% at pH 5.0. The Dox release achieved adsorption and desorption equilibrium in the solutions, which suggested that the release of Dox was pH‐sensitive. This result may be due to faster nHAP dissolution and amino group protonation in the acidic microenvironment. As reported, the stem cell microenvironment was neutral (pH = 7.4), while the tumor microenvironment was mildly acidic with an endosome pH of 6.5 and a lysosome pH of 4.0–5.0.^[^ ^24^ ^]^ Therefore, the pH‐responsive release behavior of H@C@D should reduce the toxicity of Dox to normal tissues and promote targeted drug release in the acidic tumor microenvironment.

### 2.2 Biocompatibility of Living Materials Constructed with hADSCs

**Figure 2:**
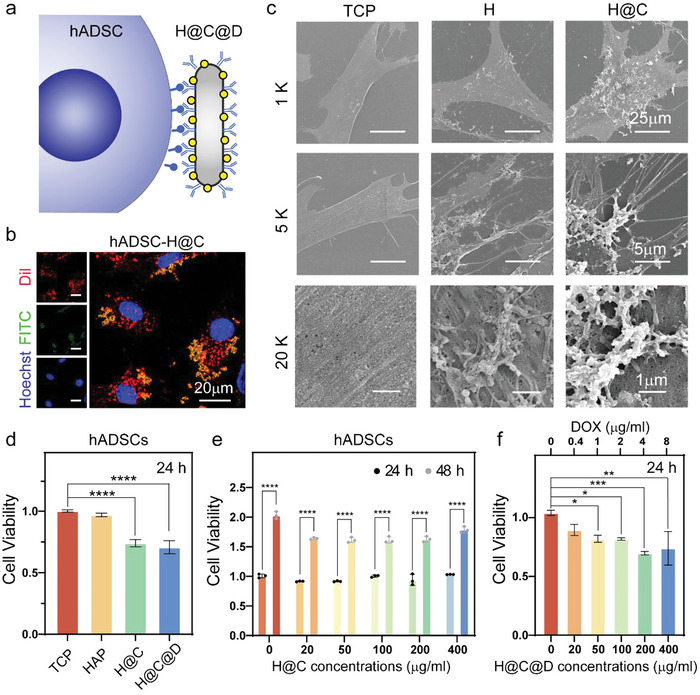
Construction and biocompatibility of the living material. a) Scheme of the living materials constructed with hADSCs and H@C@D. b) Bioconjugation of 100 µg mL^−1^ H@C on the hADSC membrane after 30 min. Red, green and blue channels show Dil‐stained cell membranes, FITC‐labeled H@C, and Hoechst‐stained nuclei, respectively. c) SEM images of hADSCs cultured with 100 µg mL^−1^ nHAP and H@C for 2 h. d) CCK‐8 assay of hADSCs cultured with 100 µg mL^−1^ nHAP, H@C, and H@C@D for 24 h. Data are presented as the mean ± SD. The *p* values were calculated using one‐way ANOVA with Bonferroni's comparison test (*n* = 3; *p < 0.05, **p < 0.01, ***p < 0.001, and ****p < 0.0001). e) CCK‐8 assay of hADSCs cultured with different concentrations of H@C for 24 and 48 h. Data are presented as the mean ± SD. The *p* values were calculated using two‐way ANOVA with Bonferroni's comparison test (*n* = 3; *p < 0.05, **p < 0.01, ***p < 0.001, and ****p < 0.0001). f) CCK‐8 assay of hADSCs cultured with different concentrations of H@C@D for 24 h. Data are presented as the mean ± SD. The *p* values were calculated using one‐way ANOVA with Bonferroni's comparison test (*n* = 3; *p < 0.05, **p < 0.01, ***p < 0.001, and ****p < 0.0001).

The living materials constructed by hADSCs and H@C@D are shown in **Figure** **2**a. H@C@D was specifically recognized by the CD44 antigen on the membrane of hADSCs. Fluorescent images captured by confocal laser scanning microscopy (CLSM) demonstrated that the FITC‐labeled H@C quickly linked to the membrane of hADSCs within 30 min and were widespread on the membrane (Figure 2b). To further observe the spreading morphologies and surface properties of hADSCs after bioconjugation, images of hADSCs and hADSCs conjugated with 100 µg mL^−1^ nHAP and H@C were captured by SEM (Figure 2c and Figure S1b,c, Supporting Information). A clear rod‐shape substance was observed on the cell surface and the EDS analysis showed that a strong Ca signal was observed on the hADSCs cultured with H@C nanorods. It indicated the attachment of H@C on the surface of hADSCs. In contrast to the control group, the morphologies of hADSCs in the H and H@C groups showed a typical shuttle shape and elongated pseudopods. The nanorod loading and antibody bioconjugation did not affect the spreading property and membrane function of hADSCs but had a positive effect on cell migration. Moreover, more nanorods were observed on the surface of hADSCs in the H@C group than in the nHAP group, which demonstrated that antibody bioconjugation facilitated the connection between nanorods and hADSCs. Additionally, the abundant nanorods on hADSCs in the H@C group after a series of dehydration processes during SEM sample preparation implied a tight connection between hADSCs and H@C. To explore in vitro biocompatibility, hADSCs were cultured with 100 µg mL^−1^ nHAP, H@C, and H@C@D for 24 h (Figure 2d). The viability of hADSCs cultured with nHAP was similar to that of the control. Antibody modification and Dox loading reduced the viability of hADSCs by ≈25.7% and 29.2%, respectively. Fortunately, all the samples of hADSCs had good proliferation conditions (Figure 2e,f). The proliferation of hADSCs was not significantly affected with various concentrations of H@C from 20 to 400 µg mL^−1^ (Figure 2e). With increased Dox loading, the proliferation of hADSCs gradually decreased by ≈33.9% compared to the control at a concentration of 200 µg mL^−1^ H@C@D (Dox loading = 4 µg mL^−1^) (Figure 2f). The proliferation of hADSCs was inhibited after 48 h at concentrations up to 400 µg mL^−1^ (Dox loading = 8 µg mL^−1^) (Figure S3, Supporting Information). These results revealed that the living material composed of 100 µg mL^−1^ H@C@D has good biocompatibility.

### 2.3 Tumor‐Tropic Ability of Living Materials

**Figure 3:**
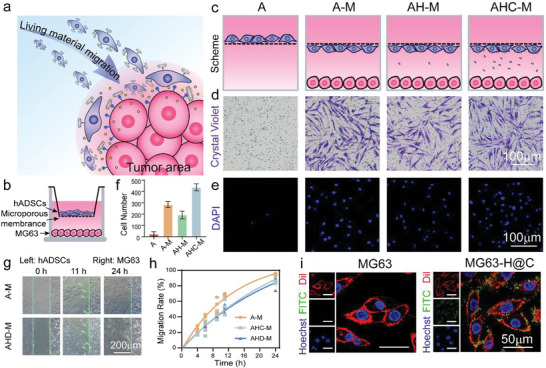
Tumor‐tropic migration of the living materials and targeted ability of H@C. a) Scheme of the migration process of the living materials. b) Scheme of Transwell construction. c) Magnification profile of both sides of the microporous membrane of the Transwell. d,e) Crystal violet staining and DAPI staining of hADSCs on the underside of the microporous membrane after 12 h of incubation. f) Statistical analysis of the number of migrated hADSCs based on DAPI staining in the 3D Transwell experiment. g) Optical images of the migration state of hADSCs and hADSCs conjugated with 100 µg mL^−1^ H@C@D for 0, 11, and 24 h. h) Migration rate of hADSCs conjugated with 100 µg mL^−1^ H@C and H@C@D in the 2D scratch model. i) Attachment of 100 µg mL^−1^ H@C on the MG63 cell membrane after 2 h. Red, green, and blue indicate Dil‐stained cell membrane, FITC‐labeled H@C, and Hoechst‐stained nuclei.

As a potential candidate for delivery vehicles, the hADSCs of living materials can migrate to the tumor area, and H@C@D is expected to target MG63 (**Figure** **3**a). To validate the tumor‐tropic ability of hADSCs and different living materials, crystal violet staining and 4′,6‐Diamidino‐2‐phenylindole (DAPI) staining were performed in a customized Transwell system. The Transwell system consisted of two chambers, in which hADSCs were seeded on the microporous membrane of the upper chamber and MG63 cells were cultured in the lower chamber (defined as the “A–M” group) (Figure 3b). Similarly, the “A” group represents the individual culture of hADSCs in the top chamber without MG63 cells cultured at the bottom. The “AH–M” group, “AHC–M” group, and “AD–M” group represent hADSCs loaded with unmodified nHAP, H@C, and H@C@D, respectively, cultured in the top chamber with MG63 cells cultured in the bottom chamber (Figure 3c). Almost no cells crossed the membrane in the “A” group, whereas many hADSCs crossed the membrane in the “A–M” group, demonstrating good tumor tropism of hADSCs (Figure 3d,e). Moreover, the percentage of hADSCs that crossed the membrane decreased by ≈32.2% after co‐culturing with 100 µg mL^−1^ unmodified nHAP. Interestingly, the bioconjugation of H@C to hADSCs promoted the migration of hADSCs with ≈54.1% more hADSCs observed (Figure 3f).

To directly observe the migration activity of hADSCs, hADSCs and MG63 cells were cultured in the bilateral chamber of the Culture‐Insert 2 Well. When the Culture‐Insert 2 Well was removed, a scratch of 500 µm was observed. After 11 h of co‐culturing, hADSCs tended to cross the scratch to the tumor side, corresponding to the tumor tropism of hADSCs. Notably, H@C‐loaded hADSCs and H@C@D‐loaded hADSCs occupied the scratch area and crossed to the tumor side for 24 h (Figure 3g and Figure S4, Supporting Information). The results also indicated that ≈87.5% and 83.5% of scratch areas were occupied by migrated hADSCs loaded with H@C and H@C@D for 24 h, respectively (Figure 3h). These results provided evidence for the good tumor tropism of hADSCs with and without nanoformulations. Furthermore, fluorescent images showed that the H@C nanorods were located on the cell membrane, which indicated that H@C specifically recognized and bound to the CD44 antigen on the membrane of MG63 cells (Figure 3i). Therefore, these findings suggested that the H@C nanorods will sequentially target the tumor after targeted delivery by the stem cell vehicle, further reducing the off‐target toxicity.

### 2.4 Synergistic Antitumor Ability of Antibody‐Modified nHAP and Dox In Vitro

**Figure 4:**
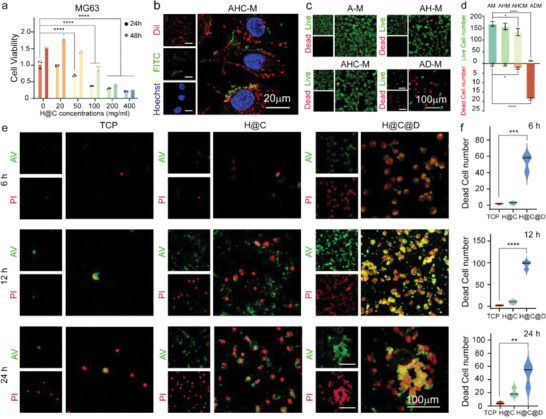
The synergistic antitumor ability of the antibody‐modified nHAP and Dox as indicated by MG63 cell apoptosis. a) CCK‐8 assay of MG63 cells cultured with different concentrations of H@C for 24 and 48 h. Data are presented as the mean ± SD. The *p* values were calculated using two‐way ANOVA with Bonferroni's comparison test (*n* = 3; *p < 0.05, **p < 0.01, ***p < 0.001, and ****p < 0.0001). b) Colocalization of H@C and MG63 cell membrane after 24 h. H@C detached from the living material. Red, green, and blue indicate the Dil‐stained cell membrane, FITC‐labeled H@C, and Hoechst‐stained nuclei. c) Live/dead staining of MG63 cells after co‐culture with the living materials for 12 h in a Transwell system. The concentration of nanorods contained in the living materials was 100 µg mL^−1^. d) Live and dead cell numbers were determined by live/dead staining of MG63 cells after co‐culture with the living materials for 12 h in a Transwell system and analysis by ImageJ. The *p* values were calculated using one‐way ANOVA with Bonferroni's comparison test (*n* = 3; *p < 0.05, **p < 0.01, ***p < 0.001, and ****p < 0.0001). e) AV/PI staining of MG63 cells after culture with 100 µg mL^−1^ H@C and H@C@D for 6, 12, and 24 h. Apoptotic cells were stained green with AV‐Fluor 488, and dead cells were stained red with PI. f) Dead cell numbers as determined by AV/PI staining of MG63 cells and analyzed by ImageJ. The *p* values were calculated using one‐way ANOVA with Bonferroni's comparison test (*n* = 3; *p < 0.05, **p < 0.01, ***p < 0.001, and ****p < 0.0001).

To assess the antitumor ability of the living materials, the toxicity of antibody‐modified nHAP was first studied. As reported, nHAP nanoparticles inhibit metabolism and induce apoptosis in various tumor cells.^[^ ^22a^ ^]^ Thus, the viability of MG63 cells cultured with various concentrations of H@C nanorods was analyzed by a cell counting kit‐8 (CCK‐8) assay (**Figure** **4**a). After 24 h of co‐culturing, the viability of MG63 cells significantly decreased at H@C concentrations greater than 50 µg mL^−1^. Of note, the cell viability decreased by ≈64.1% at a H@C concentration of 100 µg mL^−1^ compared to the control. After 48 h of culturing, the decreasing trend of cell viability was more obvious. There was no clear proliferation after treatment with H@C at the concentrations of 200 and 400 µg mL^−1^, which indicated the antitumor ability and dose‐dependent toxicity of H@C nanorods. To further determine the antitumor ability of the living materials, hADSCs were conjugated with H@C and co‐cultured with MG63 cells in a Transwell system. After 24 h of culture, the MG63 cell membranes and FITC‐labeled H@C nanorods were detected in the lower chamber of the Transwell, which suggested that H@C detached from hADSCs in the MG63 culture environment. Moreover, the FITC‐labeled H@C nanorods were distributed around the cell membrane of MG63 cells, which indicated the targeting property of H@C nanorods to MG63 cells (Figure 4b). Live/dead staining of MG63 cells was performed to observe the survival and death of tumor cells (Figure 4c,d and Figure S5, Supporting Information). Many live MG63 cells were observed in the “M” group and “A–M” group, which indicated that the migrated hADSCs had little effect on the survival of MG63 cells. Moreover, the MG63 cell density was decreased in the “AH–M” group and “AHC–M” group. The density of live MG63 cells was decreased by 92.8% in the “AD–M” group compared to the control, and many dead cells were identified, which suggested that Dox was successfully delivered to the tumor and synergistically killed MG63 cells with H@C nanorods. The apoptosis of MG63 cells was evaluated by Annexin V (AV) and PI staining (Figure 4e,f). After 6 h of culture, dead MG63 cells were detected, especially in the H@C@D nanorod group. After 12 h of culture, the number of dead MG63 cells increased by ≈4.8‐ and 47.5‐fold in the H@C and H@C@D groups compared to the control group, respectively. Additionally, the green fluorescence of AV was detected after 6 h of culture with H@C nanorods and H@C@D nanorods, and the green fluorescence of AV significantly increased after 12 and 24 h of culture (Figure S6, Supporting Information). These results confirmed the antitumor effect of H@C nanorods as detected by the induction of MG63 cell apoptosis, and Dox loading improved the antitumor efficiency. The synergistic effect of H@C nanorods and Dox in the proposed living materials may result in a highly efficient tumor killing ability.

**Figure 5:**
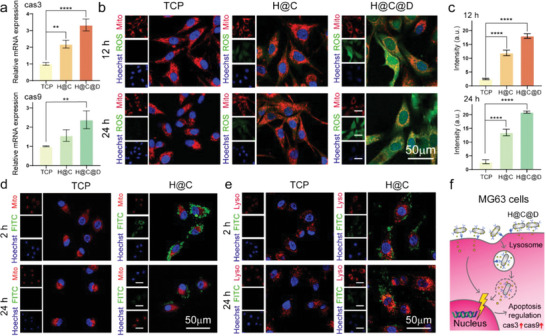
The antitumor effect and process of the proposed living materials in vitro. a) qRT–PCR analysis of apoptosis‐related genes in MG63 cells. Data are presented as the mean ± SD. The *p* values were calculated using one‐way ANOVA with Bonferroni's comparison test (*n* = 3; *p < 0.05, **p < 0.01, ***p < 0.001, and ****p < 0.0001). b,c) Mito/ROS staining of MG63 cells and the statistical analysis of the mean ROS fluorescence intensity in the cells. The red, green, and blue colors represent the mitochondria, ROS, and Hoechst‐stained nuclei, respectively. Data are presented as the mean ± SD. The *p* values were calculated using one‐way ANOVA with Bonferroni's comparison test (*n* = 3; *p < 0.05, **p < 0.01, ***p < 0.001, and ****p < 0.0001). d) Colocalization of FITC‐labeled H@C nanorods and mitochondria in MG63 cells after 2 and 24 h of culture. The red, green, and blue colors represent mitochondria, FITC‐labeled H@C nanorods, and Hoechst‐stained nuclei, respectively. e) Colocalization of FITC‐labeled H@C nanorods and lysosomes in MG63 cells after 2 and 24 h of culture. The red, green, and blue colors represent lysosomes, FITC‐labeled H@C nanorods, and Hoechst‐stained nuclei, respectively. f) Scheme of the mechanism by which H@C@D nanorods regulate the apoptosis of MG63 cells.

Induction of apoptosis is mediated through activation of initiator caspases and completed by executioner caspases, such as caspase 3 and caspase 9.^[^ ^25^ ^]^ The expression of apoptosis‐related genes was measured by quantitative real‐time polymerase chain reaction (qRT–PCR) (**Figure** **5**a). After 12 h of culture, the mRNA expression of caspase 3 in MG63 cells cultured with 100 µg mL^−1^ H@C and H@C@D nanorods was increased by ≈2.2‐fold and 3.3‐fold compared to the control, respectively. The mRNA expression of caspase 9 in the H@C and H@C@D groups was increased by ≈1.6‐fold and 2.4‐fold compared to the control, respectively. Additionally, the protein expression of caspase 3 was measured, and the results were consistent with the PCR results (Figure S7, Supporting Information). As reported, cell apoptosis is highly related to the generation of reactive oxygen species (ROS), which induce oxidative stress and direct DNA damage.^[^ ^26^ ^]^ Considering that ROS are mainly derived from cell mitochondria (Mito),^[^ ^27^ ^]^ Mito and ROS localization staining was performed (Figure 5b). After 12 and 24 h of culture, ROS were detected in MG63 cells, and they colocalized with mitochondria after culture with H@C nanorods. The ROS intensities were increased by ≈4.7‐fold and 4.8‐fold after culture with H@C nanorods for 12 and 24 h of culture, respectively, compared to the control (Figure 5c). These results supported the antitumor ability of H@C nanorods. Moreover, in the H@C@D group, the ROS intensities were further enhanced to ≈7.2‐fold and 7.5‐fold after culture for 12 and 24 h, respectively, compared to the control. The results confirmed that ROS play an important role in nanoformulation‐induced cell apoptosis. The metabolism of H@C nanorods was studied by the colocalization analysis of nanorods with the cell membrane, mitochondria, and lysosomes. The H@C nanorods showed strong yellow luminescence specifically linked to the membrane of MG63 cells after 2 h of culture, while the luminescence intensity decreased after 24 h of culture. These results implied the separation of nHAPs from MG63 cells and endocytosis of nHAPs by MG63 cells (Figures S8 and S9, Supporting Information). The separation is attributed to amino group protonation of the antibody in the acidic microenvironment, which may result in accumulation of nHAPs at lesions, thereby benefiting osteogenesis regulation of hADSCs. As shown in Figure 5d, green luminescence was detected in the H@C group after 2 and 24 h of culture, but no obvious yellow luminescence was identified even after 24 h of culture, which indicated a weak interaction between the internalized H@C nanorods and mitochondria. As shown in Figure 5e, there was a higher intensity of yellow luminescence due to colocalization between H@C nanorods and lysosomes after 24 h of culture compared to 2 h of culture, which indicated that the metabolism of nHAPs occurred in lysosomes. The proposed drug delivery and metabolism of H@C nanorods are illustrated in Figure 5f. The surface unloading of Dox and metabolism of internalized nanoformulations are the main routes to achieve Dox delivery. Because Ca^2+^ overload in lysosomes also induces apoptosis of MG63 cells, Dox and nHAP nanoformulations possess a synergistic effect on MG63 cell killing.

### 2.5 Promotion of Osteogenic Differentiation of hADSCs by Antibody‐Modified HAP Nanorods

**Figure 6:**
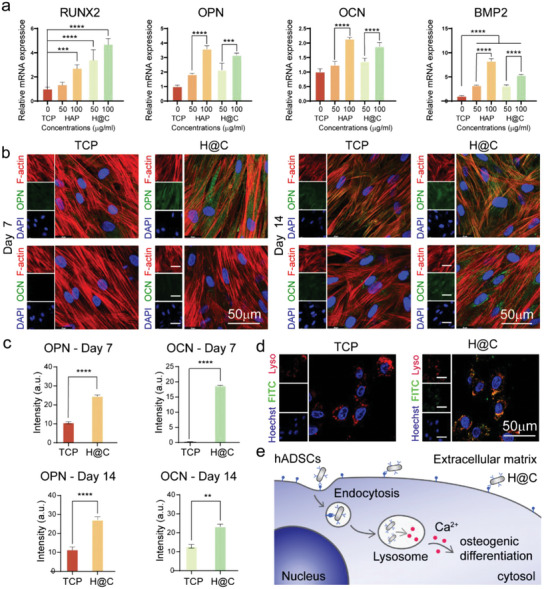
Osteogenic differentiation of hADSCs treated with H@C nanorods. a) qRT–PCR analysis of osteogenesis‐related gene expression after culture with various concentrations of HAP nanorods and H@C nanorods for 7 days. The *p* values were calculated using one‐way ANOVA with Bonferroni's comparison test (*n* = 3; *p < 0.05, **p < 0.01, ***p < 0.001, and ****p < 0.0001). b) Immunofluorescence staining of osteogenic markers after culture with 100 µg mL^−1^ H@C nanorods for 7 and 14 days. c) Mean fluorescence intensity after culture of MG63 cells with 100 µg mL^−1^ H@C nanorods for 7 and 14 days. The *p* values were calculated using one‐way ANOVA with Bonferroni's comparison test (*n* = 3; *p < 0.05, **p < 0.01, ***p < 0.001, and ****p < 0.0001). d) Colocalization of FITC‐labeled H@C nanorods and lysosomes in hADSCs after culture for 24 h. The red, green, and blue colors indicate LysoTracker‐stained lysosomes, FITC‐labeled H@C (concentration = 100 µg mL^−1^), and Hoechst‐stained nuclei, respectively. e) Scheme of the mechanism by which H@C nanorods regulate the fate of hADSCs.

The promotion of osteogenic differentiation of hADSCs by the living materials was studied in vitro by qRT–PCR (**Figure** **6**a). RUNX2 is a key transcription factor that regulates numerous genes associated with osteogenic differentiation; thus, the upregulation of RUNX2 is early evidence of osteogenic differentiation.^[^ ^28^ ^]^ After culture with 50 µg mL^−1^ nHAP and H@C nanorods for 7 days, the relative mRNA expression of RUNX2 was increased by ≈1.4‐fold and 3.4‐fold in hADSCs compared to the control, respectively. Increasing the nHAP concentration to 100 µg mL^−1^ resulted in further enhancement of expression by ≈2.7‐fold and 4.7‐fold compared to the control, respectively. Further differentiation of hADSCs is marked by osteopontin (OPN), which is a mid‐stage osteogenic marker representing the start of mineralization.^[^ ^29^ ^]^ The relative expression of OPN mRNA also showed a dose‐dependent increase with expression levels ≈3.6‐fold and 3.2‐fold higher in cells cultured with 100 µg mL^−1^ nHAP and H@C nanorods compared to the control cells, respectively. As a recently identified marker of osteogenic differentiation,^[^ ^23^ ^]^ osteocalcin (OCN) showed a similar dosage‐dependent increasing trend. However, the expression levels of OCN genes were increased by only ≈2.1‐fold and 1.9‐fold in cells cultured with 100 µg mL^−1^ nHAP and H@C nanorods, respectively, due to the late expression property. Bone morphogenetic protein 2 (BMP2) is a well‐known osteogenic differentiation factor.^[^ ^30^ ^]^ After culture with 50 and 100 µg mL^−1^ nHAP nanorods, the relative expression of BMP2 mRNA was increased by ≈3.2‐fold and 8.2‐fold that of the control, respectively. Similarly, the BMP2 expression levels were increased by ≈3.2‐fold and 5.3‐fold in hADSCs cultured with 50 and 100 µg mL^−1^ H@C nanorods, respectively. These results confirmed the positive effect of nHAPs on the osteogenesis of hADSCs, and they were consistent with the ALP activity and alizarin red staining results (Figure S10, Supporting Information). Furthermore, the immunofluorescence staining of OPN and OCN in the hADSCs after 7 and 14 days of culture with 100 µg mL^−1^ H@C nanorods was observed by CLSM (Figure 6b,c and Figure S11, Supporting information). The luminescence related to OPN protein expression was ≈2.4‐fold higher in hADSCs cultured with H@C nanorods compared to control hADSCs at Day 7, which suggested that H@C nanorods significantly improved the expression of OPN protein. There was no significant change in OPN protein expression in either group after 14 days of culture. OCN protein expression was too weak to be detected at Day 7 in the control, but OCN protein expression was detected in hADSCs cultured with H@C nanorods at Day 7. After 14 days of culture, the related luminescence of both groups increased. The protein expression of OCN in the H@C group was increased by ≈1.85‐fold compared to the control, which was consistent with the late‐stage protein expression of OCN in osteogenic differentiation. These results further confirmed the promoting property of H@C nanorods during the osteogenesis of hADSCs. Moreover, colocalization of FITC‐labeled H@C nanorods and lysosomes in hADSCs was observed after culture for 24 h (Figure 6d), which revealed the internalization of H@C nanorods by hADSCs and the location of lysosomes. The acidic environment in lysosomes would benefit the metabolism of nHAPs and the release of Ca^2+^, which could activate the calcium signaling pathway and further promote the osteogenic differentiation of stem cells (Figure 6e).^[^ ^31^ ^]^

### 2.6 Migration and Antitumor Effects of the Living Materials in the MG63 Tumor Spheroid Model

**Figure 7:**
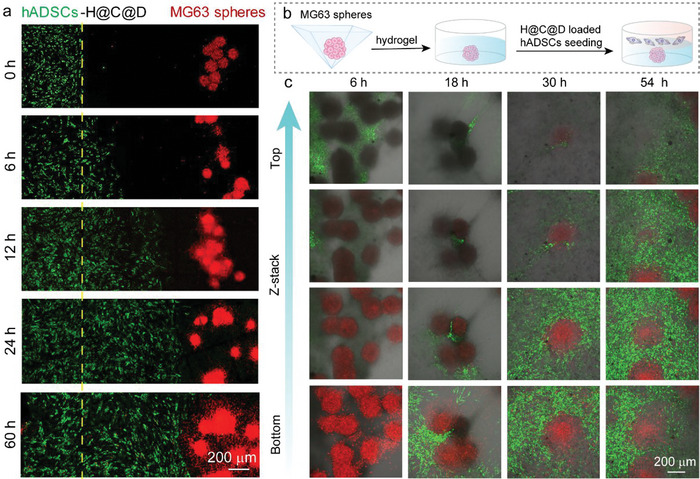
In vitro validation of the migration ability of the living materials in the MG63 tumor spheroid model. a) Left to right 3D migration assay based on the Transwell assay. H@C@D‐loaded hADSCs were labeled with pkh67 (green), and MG63 spheroids were labeled with pkh26 (red). b) Scheme of the top to bottom 3D migration model. c) Top to bottom 3D migration assay. assay. H@C@D‐loaded hADSCs were labeled with pkh67 (green), and MG63 spheroids were labeled with pkh26 (red).

An MG63 tumor spheroid model was proposed to simulate the microenvironment of osteosarcoma after surgery. First, we conducted a left‐to‐right migration assay by using a Culture‐Insert 2‐Well, in which MG63 cells aggregated into cell spheroids. The MG63 spheroids were cultured on the right side of the well in hydrogel, while the living materials were seeded on the left side (**Figure** **7**a). The living materials migrated toward MG63 spheroids from left to right, and the living materials contacted the tumor spheroids at 24 h, which was consistent with the 2D migration result (Figure 3g). To observe the all‐round migration process of the living materials, we conducted a top‐to‐bottom migration assay (Figure 7b). After culture for 6 h, the living materials were mainly detected at the top hydrogel layer (Figure 7c and Figure S12, Supporting Information). Up to 18 h, the living materials were observed around the MG63 spheroids at the top, middle, and bottom layers. After long periods of culture, the living materials nearly enclosed the MG63 spheroids. This enclosure state of the living materials around the tumor cells would facilitate efficient targeted delivery of the antitumor drug.

The ROS levels in the MG63 spheroids were then detected in the 3D MG63 tumor spheroid model. After culture for 6 h, few ROS were observed in the MG63 spheroids on the bottom layer. Because most of the living materials aggregated on the top layer, ROS were induced by the released Dox in the hydrogel. Subsequently, a large amount of ROS was detected in the MG63 spheroids after culture with the living materials for 24 and 48 h. Contact between hADSCs and MG63 cells was clearly observed. In contrast, barely any ROS were observed in the groups of individual MG63 spheroids and MG63 spheroids co‐cultured with pure hADSCs (Figures S13 and S14, Supporting information). Additionally, ROS were mainly detected in the spheroids instead of the H@C@D‐loaded hADSCs, which provided additional evidence for the low toxicity of the nanoformulations on hADSC vehicles. Together, these results confirmed the migration ability and antitumor effect of the living materials in a 3D microenvironment.

### 2.7 Bone Regeneration and Antitumor Effects of the Living Materials In Vivo

**Figure 8:**
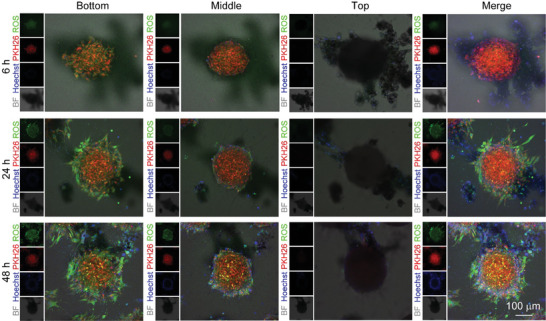
In vitro validation of the antitumor effect of the living materials in a 3D model. Tumor spheroids were labeled with PKH26 (red), and the nuclei of hADSCs and tumor spheroids were stained blue with Hoechst. ROS were stained green. The different layers of MG63 spheroids were observed by CLSM.

**Figure 9:**
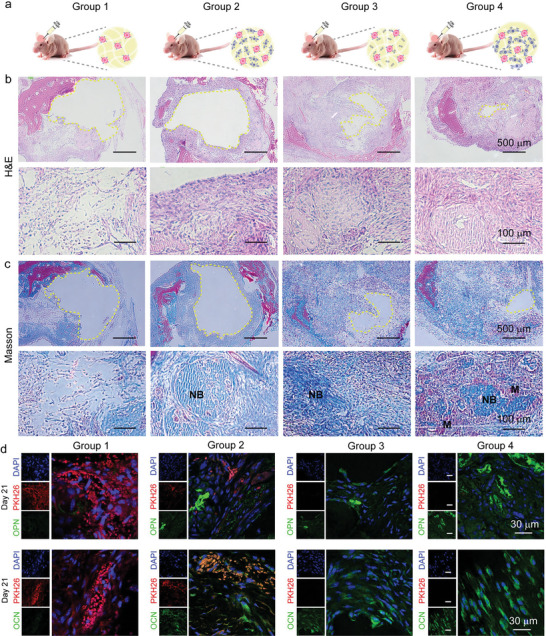
In vivo validation of the bone regeneration and antitumor effect of the living materials in nude mouse. a) The scheme of animal experiment. The nude mouse was injected with Matrigel contained with tumor cells (Group 1), tumor cells and hADSCs (Group 2), tumor cells and H@C@D nanorods (Group 3), and tumor cells and the living materials (Group 4). b) H&E staining images of calvarial bone defect sections at day 14. The defect area is marked with a yellow dotted line. c) Masson‐trichrome staining images of calvarial bone defect sections at day 14. The defect area is marked with a yellow dotted line. d) Immunostaining of tissue slices for the osteogenic markers and fluorescent images of PKH26‐labeled tumor cells after 21 days. Cell nuclei were stained with DAPI (blue), OCN and OPN are shown in green, while tumor cells are in green.

To further assess the bone regeneration and antitumor effect of the living material in the scenario of osteosarcoma after surgical resection, a subset of tumor cells were injected into the bone defect area in nude mice, followed by the injection of stem cells, H@C@D nanorods, and living materials, respectively (**Figure** **9**a). Tissue samples were collected at 7, 14, and 21 days, respectively, as shown in Figure S15, Supporting Information. Histological analyses were performed to evaluate the regenerative states of the bone defect area using H&E and Masson's trichrome staining (**Figure** **8**b,c and Figure S16, Supporting Information). No inflammatory cells were observed in the H&E staining. The defect areas of the bones were reduced obviously with H@C@D nanorods (group 3) and living materials (group 4) compared to the control (group 1) and stem cells group (group 2) at 14 days. More newly formed bone tissues and mature bone marrow were observed in Group 3 and 4 than the other groups. It indicated that the H@C@D nanorods have good bone regeneration ability in short time and the dox loading would not affect the regeneration ability. The best bone regeneration in group 4 indicated the synergistic effect of H@C@D nanorods and stem cells in the living materials. It is notable that the individual injection of stem cells had little effect on bone formation, which may be attributed to the existence of residual tumor cells due to the limited antitumor ability of individual stem cells. To examine this hypothesis, PKH26 (red dye)‐labeled tumor cells were tracked during a long period of regeneration process (Figure 9d and Figure S17, Supporting Information). After 21 days, a large amount of tumor cells were observed in the control and stem cells groups. On the contrary, no tumor cells were detected in the H@C@D group and the living materials group. It demonstrated that the dox loading nanorods effectively inhibit the proliferation of the tumor cells. But the limited anti‐tumor ability of stem cells could not inhibit the proliferation of tumor cells after a long‐term feeding (Figure S17, Supporting Information). The higher vitality of tumor cells would result in the domination of tumor cells after long‐term co‐culture with stem cells. In addition, the expression of OPN and OCN proteins in the defect areas were also assessed. After 21 days, higher OPN and OCN protein expression were detected in the living material group. It indicated the good therapeutic effect of the living materials with both strong inhibition of tumor cells and excellent bone regeneration properties.

## 3 Conclusion

In summary, we engineered living materials with tumor‐targeting ability to inhibit osteosarcoma after surgery and regenerative ability for bone tissue. The living materials are composed of hADSCs and Dox‐loaded Anti‐CD44 antibody‐modified hydroxyapatite nanorods (H@C@D nanorods). The hADSCs play the roles of active vehicle and stem cells for osteogenesis. The nanoformulation of H@C@D nanorods contributes to the further targeted delivery of Dox and nHAPs due to antibody modification. The combination of Dox and nHAPs induces apoptosis of MG63 cells, attributed to ROS accumulation and the activation of caspase 3 and caspase 9. The tumor spheroid model and in vivo animal model further confirmed the targeted delivery, killing ability, and regenerative ability of the living materials. Therefore, the engineered living materials provide new opportunities for targeted tumor therapy, bone regeneration, and other therapeutic applications.

## 4 Experimental Section

### Chemicals

For material synthesis, 1‐octadecylamine, oleic acid, ethanol, Ca(NO_3_)_2_, Na_3_PO_4_, (3‐aminopropyl) triethoxysilane (APTES), ammonia, hydrochloric acid (HCl), sodium hydroxide (NaOH) were purchased from Sinopharm Chemical Reagent Co., Ltd. Dox. *N*‐Hydroxysulfosuccinimide sodium salt (Sulfo‐NHS) was purchased from Shanghai Macklin Biochemical Co., Ltd. Phosphate buffered saline (PBS), EDC, and Dox were purchased from Sigma‐Aldrich Chemical Reagent Co., Ltd. Anti‐CD44 antibody was purchased from BioLegend (America)

For cell experiments, minimum Essential Medium (MEM), *α*−minimum essential medium (*α*−MEM), fetal bovine serum (FBS), and Penicillin–Streptomycin solution (P/S) were purchased from Gibco (America). Cell Plasma Membrane Staining Kit with DiI (Red Fluorescence), Hoechst33342, propidium iodide (PI), and calcein acetoxymethyl ester (Calcein‐AM) were purchased from Beyotime Biotechnology Co., Ltd (China). DAPI was purchased from Abcam. CCK‐8 was purchased from Dojindo (Japan). Crystal violet staining solution and Annexin V‐kFluor488/PI staining assay were purchased from KeyGEN BioTECH (China).

### Cell Culture

hADSCs were extracted from adipose tissue of a male healthy donor (≈30 years old) after obtaining informed written consent from the participant. The study was approved by the Research Ethics Committee of Qilu Hospital of Shandong University (Project No. KYLL‐2019(KS)‐086). hADSCs were cultured in *α*‐MEM medium supplemented with 10% FBS and 1% P/S, and maintained in a humidified atmosphere of 5% CO_2_ at 37 °C. MG63 cells which were derived from a 14 years old white male with osteosarcoma were purchased from ICell Bioscience Inc (China) and cultured in MEM medium supplemented with 10% FBS and 1% P/S under a humidified atmosphere of 5% CO_2_ at 37 °C. Both the culture media were replaced every 2 days. For osteogenic differentiation of hADSCs, hADSCs were cultured in osteogenic differentiation medium (basal medium plus 1 × 10^−8^ m dexamethasone, 1 × 10^−2^ m *β*‐glycerophosphate, and 5 ×10^−5^ l‐ascorbic acid). The culture medium was replaced every 2 days.

### Synthesis of HAP Nanorods

nHAP were prepared by hydrothermal method. Briefly, 1‐octadecylamine (0.5 g) was dissolved in oleic acid (4 mL) in 60 °C under stirring for 30 min. Then, ethanol (16 mL) was added in the mixture and Ca(NO_3_)_2_ (0.28 m, 7 mL) dissolved in ultrapure water was added dropwise while stirring. Subsequently, Na_3_PO_4_ (0.168 m, 7 mL) was added dropwise and stirred for 10 min. Then, the mixture was moved into a 50 mL Teflon lined autoclave, and heated at 180 °C for 12 h. Finally, the obtained nanorods deposited at the bottom of autoclave and washed with ethanol and ultrapure water in a centrifuge at 10 000 rpm for three times.

### Antibody Bioconjugation

To connect with antibody, nHAP were first modified with amino group. Typically, nHAP (100 mg) were dispersed in 10 mL of ethanol/water mixtures (9/1, v/v) under sonication. Then, APTES (100 µL) was successively added into the above mixture and ammonia was used to adjust the pH value of the mixture to 10 under vigorous stirring for 2 h. Finally, the obtained nHAP‐NH_2_ were collected by centrifugation at 10 000 rpm and washed with ethanol to remove unreacted APTES. For antibody bioconjugation, Anti‐CD44 antibody (50 µL, 0.2 mg mL^−1^) was added in ultrapure water (1 mL) and HCl solution (0.1 m) was used to adjust pH to 5.8. Then, EDC (400 µL, 0.2 g mL^−1^) and Sulfo‐NHS (400 µL, 0.2 g mL^−1^) were successively added to the above mixture and incubated for 30 min at room temperature. Subsequently, NaOH was used to adjust the pH value of mixture to 7, following with addition of nHAP‐NH_2_ and incubated overnight at 4 °C. The generated Anti‐CD44 antibody containing nHAP were collected by centrifugation at 10 000 r min^−1^ and washed with PBS for three times.

### Observation of the Bioconjugation of H@C on hADSCs

To observe the position and distribution state of H@C on hADSCs membrane, CLSM (Leica, Germany) was first used to capture the fluorescent images. First, H@C was marked with FITC by incubating at 4 °C overnight. And 100 µg mL^−1^ of the obtained H@C‐FITC were added into the culturing medium of hADSCs for 30 min. Subsequently, the membrane of H@C‐FITC connected hADSCs were stained by Dil (Red Fluorescence) for 30 min according to the standard protocols described by the manufacturer, following with the nucleus staining by Hoechst for 10 min at 37 °C.

Furthermore, SEM was used to observe the spreading morphologies and surface properties of H@C connected hADSCs after bioconjugation. First, hADSCs were cultured with 100 µg mL^−1^ H@C on 24‐well tissue culture plate for 2 h and the samples were washed with PBS for three times. Then, 2.5% glutaraldehyde solution was used to fix the samples for 30 min, following with PBS washing step for three times. Next, the samples were dehydrated using gradient alcohol solutions (30%, 50%, 70%, 80%, 90%, 95%, 98%, and 100%) and stored in 37 °C. The samples were observed under a HITACHI S‐4800 scanning electron microscope after 50 s of Au spraying at a current of 20 µA.

### The Biocompatibility of nHAP, H@C, and H@C@D on hADSCs

To observe the cell viability of hADSCs and MG63 culturing with nHAP, H@C, and H@C@D, CCK‐8 assay was carried out according to manufacturer instruction. Typically, hADSCs were cultured in 96‐well culture plates with specific nanorods in cell proliferation medium and 10 µL of CCK‐8 solution was added to the well at specific time point. The cells incubated at 37 °C for 1 h and the level of water‐soluble formazan dye was recorded at a wavelength of 450 nm by a microplate reader (Multiscan MK3, Thermo, USA). Triplicate parallel replicates for each group were conducted.

### Cell Migration Assays

To verify the tumor‐tropic ability of living materials, the Transwell transmigration assay was carried out by using Transwell with 0.8 µm pore polycarbonate membrane insert. The Transwell system contained upper and lower chamber, separated by porous membrane of the insert. hADSCs were seeded on the mesoporous membrane of the insert culturing with *α*‐MEM based medium while MG63 were cultured at the lower chamber of Transwell culturing with MEM based medium. And nanorods were added to the inserts and incubated for 2 h. After that, the inserts contained hADSCs moved to the lower chamber cultured with MG63 and co‐cultured for 12 h. Then, crystal violet and DAPI staining was carried out to the migration ability of hADSCs. Typically, hADSCs on the lower side of the Transwell membrane were washed with PBS three times and fixed with 4% paraformaldehyde at room temperature for 20 min. For crystal violet staining, the insert was removed and immersed in crystal violet staining solution and stained for 10 min. After washing with PBS three times, the migrated cells were observed by fluorescence inverted microscopy. For DAPI staining, cells on the lower side of the inert membrane were permeabilized by 0.1% Triton X‐100 for 10 min after fixing with 4% paraformaldehyde for 20 min which was consistent with crystal violet staining. After washing with PBS three times, the samples were stained by DAPI for 5 min at room temperature and following with PBS washing step. Finally, the migrated cells were observed by fluorescence inverted microscopy.

In order to further evaluate the migration ability of hADSCs connected with H@C and H@C@D in direct contact with MG63 cells, Culture‐Inserts 2 Well (ibidi, Germany) was used to build a co‐culture assay. Typically, Culture‐Inserts 2 Well was moved to the Petri dish. After seeding the hADSCs on the left well and MG63 on the right well of the Culture Insert, the H@C and H@C@D were added in the left well and incubating for 12 h. After cell attachment, the insert can be easily removed which allows for hADSCs and MG63 to grow separately from each other, while sharing the same medium based on *α*‐MEM. Meanwhile, a scratch of 500 µm was formed after removing the culture insert and hADSCs can spontaneously cross the scratch to the tumor on the other side. Thus, the migration activity of hADSCs connected with H@C and H@C@D can be observed by microscope for continuous 24 h. Additionally, the migration rate of hADSCs can be calculated by the scratch area measured by ImageJ. Briefly, the scratch area was measured by Image J after hADSCs migrating for 0, 3, 6, 9, 11, and 24 h, and cell migration rate by following equation.

(1) $$\mathrm{Migration}\;\mathrm{rate}=\frac{\mathrm{Blank}\;\mathrm{area}\;\mathrm{without}\;\mathrm{cell}\;\mathrm{migration}}{\mathrm{Initial}\;\mathrm{scratch}\;\mathrm{area}}\times 100\%$$

### The Cytotoxicity of Various Concentrations of H@C on MG63 Cells

To assess the anti‐tumor ability of H@C nanorods, CCK‐8 assay was carried out according to manufacturer instruction. Typically, MG63 cells were cultured in 96‐well culture plates with 0, 20, 50, 100, 200, and 400 µg mL^−1^ H@C nanorods in cell proliferation medium for 24 and 48 h. Then, 10 µL of CCK‐8 solution was added to the well and the cells were incubated at 37 °C for 1 h. The level of water‐soluble formazan dye was recorded at a wavelength of 450 nm by a microplate reader (Multiscan MK3, Thermo, USA) and triplicate parallel replicates for each group were conducted.

### Verification of the Deciduous Activity of H@C from the Living Material

To observe the H@C falling from the living material, hADSCs were cultured with 100 µg mL^−1^ H@C nanorods in the Transwell insert for 2 h. Then, the basic medium was used to wash the ungrafted H@C from hADSCs. Subsequently, the insert containing the living material was moved to the upper of MG63 cells which cultured in lower chamber of Transwell. After co‐culturing for 24 h, the Dil and Hoechst staining were carried out to determine the deciduous activity of H@C from the living material. The staining steps were consistent with the staining for hADSCs described above.

### Generation of MG63 Spheroids

MG63 cell spheroids were prepared by using Aggrewell 800 (Stem Cell Tech. USA). Briefly, the 24‐microwell plate was first coated by Anti‐Adherence Rinsing solution. Then single MG63 cells suspension with cell number of 9 × 10^5^ was added to the microwell following with centrifugation. The MG63 spheroids were harvested after incubating the plate at 37 °C with 5% CO_2_ and 95% humidity for 24 h.

### Construction of 3D Migration Model

For left‐to‐right migration model, hADSCs and MG63 spheroids were separately seeded on the left and right side of Culture‐Inserts 2 Well (ibidi). Briefly, hADSCs were labeled with PKH67 (sigma) according to the manufacturer's instructions. Then, H@C@D nanorods were added into the PKH67‐labeled hADSCs suspension and incubated for 30 min at 37 °C with 5% CO_2_ and 95% humidity. After that, the nADSCs were rinsed with PBS for three times to wash off the unlinked nanorods. And the MG63 single cell suspension was labeled with PKH26 and then added to 24‐microwell plate to generate the spheroids. Then the PKH26‐labeled MG63 spheroid were dispersed in Matrigel (1 mg mL^−1^) and seeded on the right side of Culture‐Inserts while PKH67‐labeled H@C@D‐loaded hADSCs were seeded on the left. After 24 h incubation, the Culture‐Inserts were removed and the migration state was observed with CLSM at specific time point. For top‐to‐bottom migration model, the petri dish was first coated with Matrigel (2 mg mL^−1^) at 37 °C. Then the PKH26‐labeled MG63 spheroids were dispersed in Matrigel (1 mg mL^−1^) and seeded on the coated plate. After culturing at 37 °C with 5% CO_2_ and 95% humidity for 6 h, the PKH67‐labeled H@C@D‐loaded hADSCs were seeded on the top of the Matrigel layer. The migration state was observed with CLSM at specific time point.

### Mouse Calvarial Defect Surgery

The study was conducted according to protocols approved by the Ethics Committee on Scientific Research of Shandong University Qilu Hospital (Accreditation number: KYLL‐2021(ZM)‐177). Fourteen weeks old female BALB/c nude mice were used to create a defect. The nude mice were anesthetized, and a 4 mm‐diameter calvarial critical‐sized defect was created in the center of the calvarium under constant irrigation. Each defect was rinsed with saline to remove bone debris. The defect area of control mice was filled with 10^6^ MG63 cells and Matrigel. Group 2 was filled with 10^6^ MG63 cells, 10^7^ hADSCs, and Matrigel. Group 3 was filled with 10^6^ MG63 cells, 1 mg H@C@D, and Matrigel. Group 4 was filled with 10^6^ MG63 cells, 10^7^ living materials, and Matrigel. Skin incisions were sutured. Animals were observed daily, and body weights were measured weekly. Samples were harvested at 7, 14, and 21 days after surgical implantation, and fixed in 3.7% paraformaldehyde for 1 week at room temperature. Then, the samples were decalcified with 10% EDTA and sectioned for H&E staining, Masson trichrome staining, and immunostaining.

## Conflict of Interest

The authors declare no conflict of interest.

## Author Contributions

H.X. and M.H. contributed equally to this work. H.X. conceived and conducted the experiments, performed analyses, and wrote the manuscript. M.H investigated and carried out formal analysis. K.L., X.C., and Y.Y. reviewed and edited the manuscript. H.Z. contributed to the concept promotion and revise manuscript. H.L. contributed to technical and theoretical support on the experiments. J.Q., H.L., and Y.S. contributed to conceptualization, resources, supervised the research, and revised the manuscript. All authors contributed to the article and approved the submitted version.

## Supporting information

Supporting Information

## Data Availability

The data that support the findings of this study are available from the corresponding author upon reasonable request.
