# Supplementary material for: CD44 and HAP‐Conjugated hADSCs as Living Materials for Targeted Tumor Therapy and Bone Regeneration
Source: Adv Sci (Weinh). 2023 May 8;10(20):2206393. doi: 10.1002/advs.202206393 (PMC10369264; doi:10.1002/advs.202206393)
Supplement: Supplementary file 1 — Supporting Information [file ADVS-10-2206393-s001.pdf]

ADVANCED SCIENCE
Open Access

Supporting Information

for *Adv. Sci.*, DOI 10.1002/advs.202206393

CD44 and HAP-Conjugated hADSCs as Living Materials for Targeted Tumor Therapy and Bone Regeneration

He Xia, Min Hao, Kaiwen Li, Xin Chen, Liyang Yu, Jichuan Qiu, Hongyu Zhang, Haijun Li*, Yuanhua Sang* and Hong Liu*

Supporting Information of

CD44 and HAP-conjugated hADSCs as living materials for targeted tumor therapy and bone regeneration

He Xia, Min Hao, Kaiwen Li, Xin Chen, Liyang Yu, Jichuan Qiu, Hongyu Zhang, Haijun Li*, Yuanhua Sang*, Hong Liu*

Affiliations

H. Xia, M. Hao, X. Chen, L. Yu, Prof. J. Qiu, Prof. Y. Sang, Prof. H. Liu

State Key Laboratory of Crystal Materials

Shandong University

Jinan 250100, P. R. China

Email: sangyh@sdu.edu.cn (Y. Sang), jichuan.qiu@sdu.edu.cn (J. Qiu)

K. Li, H. Zhang, H. Li

Department of Geriatrics and the Key Laboratory of Magnetic Field-free Medicine & Functional Imaging (MF), Qilu Hospital, Cheeloo College of Medicine

Shandong University

Jinan 250100, P. R. China

Email: lihaijun@qiluhospital.com (H. Li)

Dox Loading and Release: Dox can be loaded onto nHAP by simple absorption and Dox loading amount was determined by the UV–vis absorbance at 482 nm. Typically, series of Dox samples (10, 20, 40, 60, 80, 100 μg/ml) were prepared and the absorbance at 482 nm was recorded by ultraviolet–visible (UV–Vis) spectroscopy (UV-9000, Shanghai Metash Instruments Co., Ltd) to establish the standard curve of Dox solution in PBS. For Dox absorption, Anti-CD44 antibody containing nHAP (10mg) were added in Dox solution (1mg/ml) under stirring for 24 h in the dark. Then, Dox-loaded anti-body containing nHAP (H@C@D) were washed with PBS for 3

times to remove the unloaded Dox. Then, the supernatant was collected to calculate the Dox absorption amount. To determine the Dox releasing ability, H@C@D were immersed in PBS with different pH values (5.0 and 7.4) and the supernatant was collected at predetermined time points to calculate the Dox releasing amount by following equation.

$$\text{Dox releasing amount } (\%) = \frac{\text{Dox absorption amount} - \text{Dox in the supernatant}}{\text{Dox absorption amount}} \times 100\%$$

Live and dead staining assay: To visually assess the anti-tumor ability of the living material, live/dead staining was performed according to the manufacturer's instructions. Briefly, hADSCs were cultured with 100 μg/mL nHAP, H@C and H@C@D nanorods in the Transwell inserts for 2 h, respectively. Then, the inserts were removed to the upper of MG63 cells which cultured in lower chamber of Transwell. After co-culturing for 12 h, MG63 cells were stained by 500 μL of MEM containing 1×10^{-6} M Calcein-AM and 6×10^{-6} M PI. After incubating for 30 min at 37 °C, the cells were washed with PBS and observed by fluorescence inverted microscopy.

PI and AV staining: To observe the apoptosis of MG63 cells culturing with H@C and H@C@D nanorods, AV-kFluor488/PI staining was carried out according to the manufacturer's instructions. Briefly, MG63 cells cultured with 100 μg/mL H@C and H@C@D nanorods for 6, 12 and 24 h. Then, 500 μL of binding buffer containing 5 μL of AV-kFluor488 and 5 μL of PI replaced the medium after washing with PBS for three times. After incubating for 5 min at 37 °C, the cells were observed by fluorescence inverted microscopy.

RT-PCR analysis: To assess the apoptosis related gene expression, MG63 cells were cultured with 100 mg/ml H@C and H@C@D nanorods for 12 h. Then, TRIzol reagent was used to extract total RNA and the concentration and purity of extracted RNA were measured by MicroDrop® spectrophotometer (BIO-DL Corporation, Shanghai, China). Subsequently, Eco M-MLVRT Kit for qPCR (accurate Biotechnology Co., Ltd, Hunan, China) was used to gain the cDNA. Finally, the gene

expression levels of caspase3 and caspase 9 were measured by a 7500 Real-Time PCR system (Applied Biosystems, Germany). Similarly, the osteogenic related gene expression was also detected. Briefly, hADSCs were cultured with various concentrations nHAP and H@C@D nanorods for 7 days. The following RNA extraction and Polymerase Chain Reaction were performed as protocol described above. All the primer sequences are provided in Table S1 (Supporting Information). Target gene expression was normalized to that of β-actin expression and presented as the Mean ± SD.

ROS staining: To assess the cellular ROS production, Reactive Oxygen Species Assay Kit (Beyotime Biotechnology Co., Ltd.) was used to detect the ROS level of MG63 cells. Briefly, MG63 cells were cultured with 100 μg/mL nHAP, H@C and H@C@D nanorods for 12 and 24 h. Then, the MG63 cells were incubated by serum-free medium containing with 2′,7′-dichlorofluorescein diacetate (DCFH-DA) for 20 mins at 37 °C. DCFH-DA can be oxidized by ROS to produce green fluorescent dichlorofluorescein (DCF). After washing with PBS twice, the cells were observed by CLSM. The protocol of ROS staining of MG63 spheroids is same as above.

Mito and Lyso staining: To distinguish whether H@C nanorods were transported to lysosomes or mitochondria after the internalization, lysosome and mitochondria staining of MG63 cells were performed. Briefly, MG63 cells were cultured with 100 μg/mL nHAP, H@C nanorods for 2 and 24 h. At specific time point, MG63 cells were washed with PBS three times. To label the lysosome, Lyso-Tracker Red (Beyotime Biotechnology Co., Ltd.) working solution was replaced the culturing medium. After incubation for 30 min at 37 °C, cells were washed with PBS. Then the nucleus was stained by Hoechst for 5 mins at 37 °C and the samples were rinsed by PBS. Finally, the cells were observed by CLSM. To label the mitochondria, Mito-Tracker Red CMXRos (Beyotime Biotechnology Co., Ltd.) working solution was replaced the culturing medium. After incubation for 20 min at 37 °C, cells were washed with PBS. Then the nucleus was stained by Hoechst for 5 mins at 37 °C and the samples were rinsed by PBS. Finally, the cells were observed by CLSM.

Immunofluorescence (IF) staining: To verify the osteogenic related protein expression, hADSCs were cultured with various concentrations of nHAP and H@C nanorods for 7 and 14 d in 24-well plates. Then, the protein expression level was observed by IF staining. Typically, hADSCs were washed with PBS and fixed with 4% paraformaldehyde at room temperature for 20 min. Then, 0.1% Triton X-100 was used to permeabilize hADSCs for 10 min, and 1% bovine serum albumin (BSA) was used to block hADSCs for 30 min at room temperature. Subsequently, the cells were incubated overnight at 4 °C with primary osteocalcin (OCN) and osteopontin (OPN) antibodies at a 1:1000 dilution in 1% BSA solution (rabbit polyAb, Proteintech). Then, the cells were incubated with the green fluorescent labeled secondary antibody at 1:200 dilution in 1% BSA solution for 1 h at room temperature. After washing three times with PBS, F-actin was stained with phalloidin (Red fluorescence) for 30 min. After washing three times with PBS, the nucleus was stained with DAPI for 5 min. Finally, the cells were observed with CLSM.

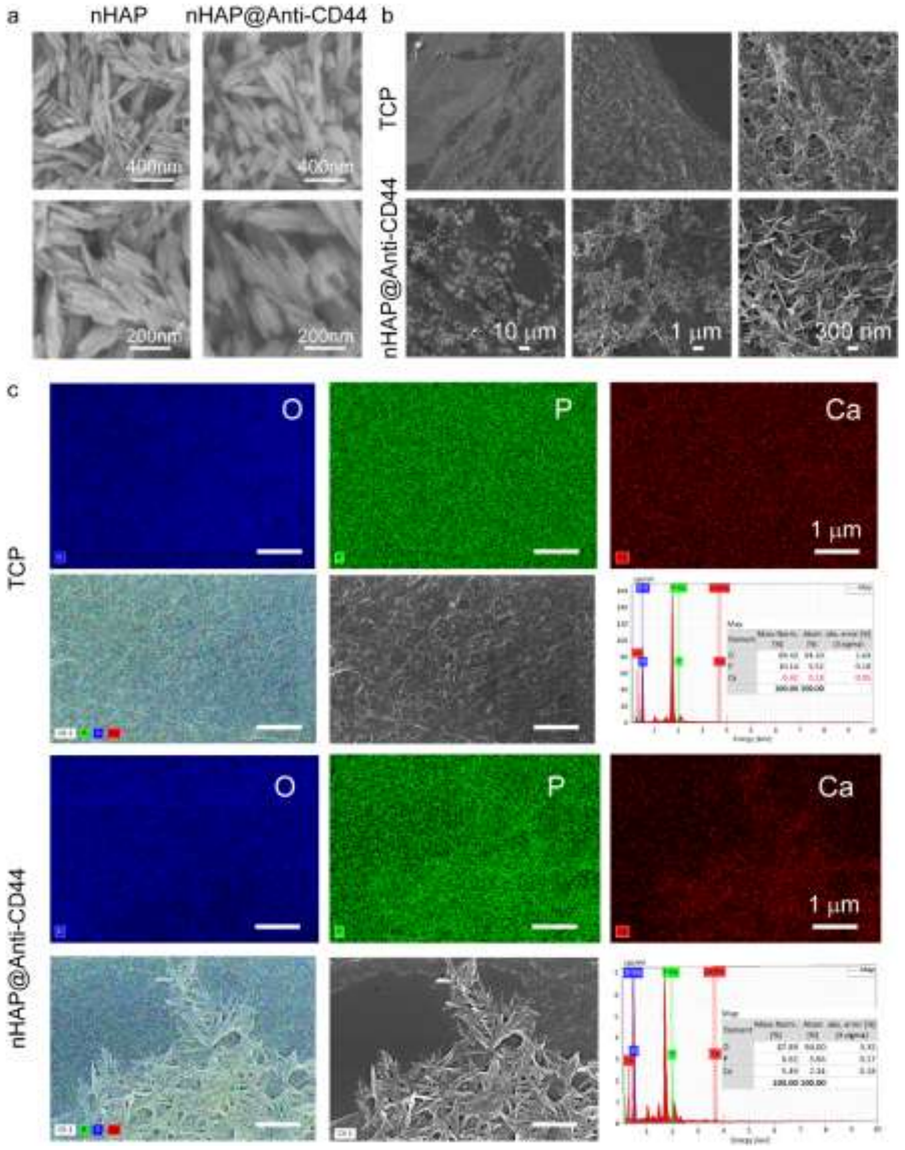

Figure S1. The morphology of the nanorods and the living materials. (a) SEM images of nHAP and nHAP@Anti-CD44 antibody (H@C). (b-c) SEM images and EDS mapping images of cell surface of hADSCs cultured with 0 and 100 μg/ml H@C for 2 h.

The SEM images of nHAP and nHAP@Anti-CD44 antibody (H@C) were captured (Figure S1a). The results showed that the antibody modification did not affect the morphology of HAP nanorods. Both the nanorods showed uniform size with a high aspect ratio which is consistent with TEM images (Figure 1b, c). Moreover, the surface of hADSCs cultured with H@C nanorods was observed by SEM.

As shown in Figure S1b, clear rod-shape substances attached to the cell surface indicated the successful attachment of H@C on the surface of hADSCs. Furthermore,

the compositional distribution of Ca, P and O in the surface of hADSCs was investigated by EDS mapping (Figure S1c). The EDS analysis showed that a strong Ca signal was observed on the hADSCs cultured with H@C nanorods. And the element content in terms of normalized mass % is shown in the insert table of Figure S1c. The mass of Ca on the surface of hADSCs cultured with H@C nanorods was 5-fold than that of hADSCs. The results confirmed the successful attachment of H@C on the surface of hADSCs.

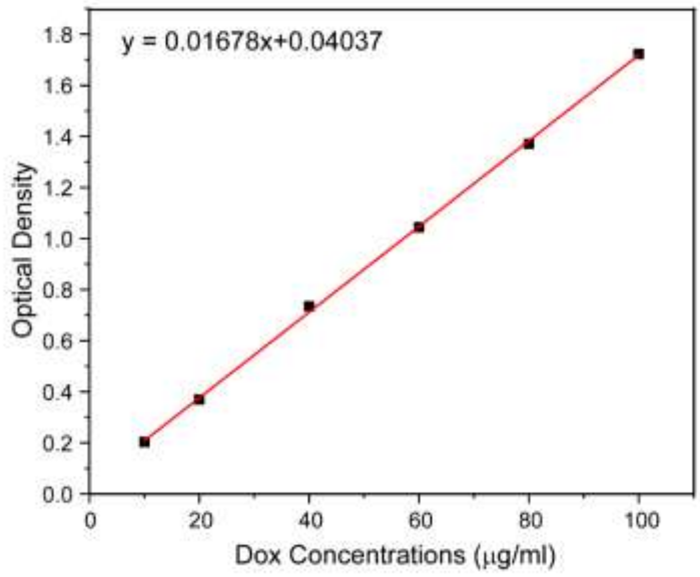

Figure S2. The Standard Curve of Dox in Phosphate-Buffered Saline (PBS).

As reported, Dox has absorption in the visible range with absorption maxima at 482 nm.[1] Therefore, a series of concentrations of standard Dox samples were prepared and the UV–vis absorbance at 482 nm was recorded to make the standard curve. The equation for the Dox standard curve is $y=0.01678x+0.04037$. When calculating the adsorption amount and releasing amount of Dox by nHAP, the Dox concentration (x) can be calculated according to the absorbance (optical density, y). Dox adsorption amount (nHAP) = Dox total amount - Dox in the supernatant (after centrifugation). Dox releasing amount (nHAP) = (Dox absorption amount - Dox in the supernatant (after centrifugation)) / Dox absorption amount.

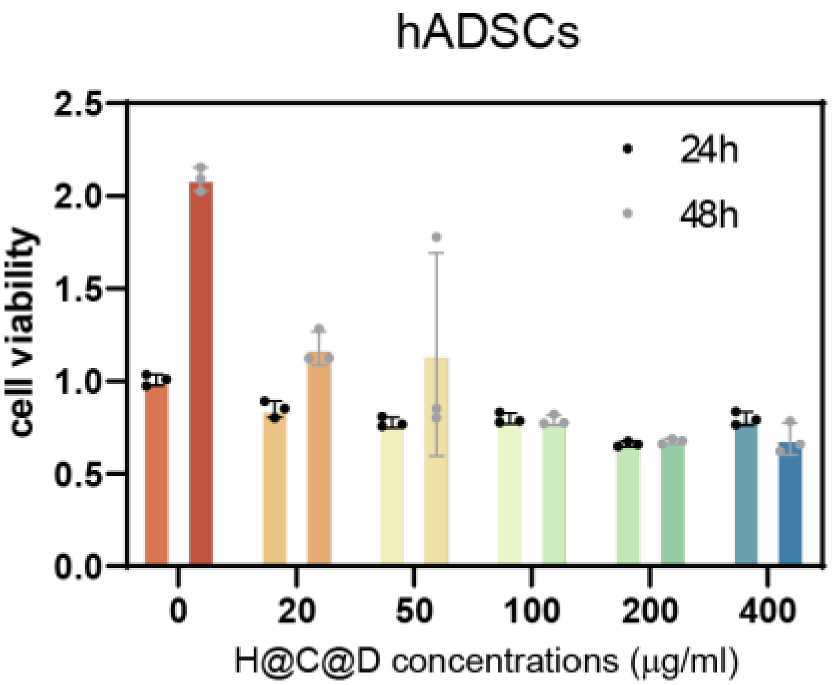

Figure S3. CCK-8 assay of hADSCs cultured with different concentrations of H@C@D for 24 h and 48 h.

As shown in Figure S3, the viability of hADSCs connected with 0-400 μg/mL H@C@D changed little for 24 h. High dose of H@C@D with concentrations above 100 μg/mL hindered the proliferation ability of hADSCs after 48h. Nevertheless, the cell viability of hADSCs cultured with 100 μg/mL H@C@D for 24 h is similar with the control, indicating that the living material composed of 100 μg/mL H@C@D have good biocompatibility and still active for payload transport.

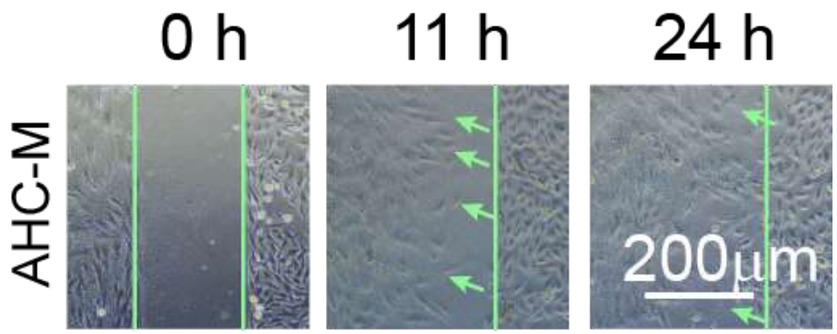

Figure S4. Optical images of the migration state of hADSCs anchored with 100 μg/mL H@C for 0, 11 and 24 h.

After seeding the hADSCs on the left well and MG63 on the right well of the Culture Insert, the H@C nanorods were added in the left well and incubating for 0, 11 and 24 h. Then, the migration process of hADSCs loaded with H@C. As shown in Figure S4, hADSCs conjugated with H@C tended to cross the scratch to the tumor on the other side after 11 h co-culturing, and most scratch area were occupied by hADSCs, indicating the good tumor tropism of H@C loaded hADSCs.

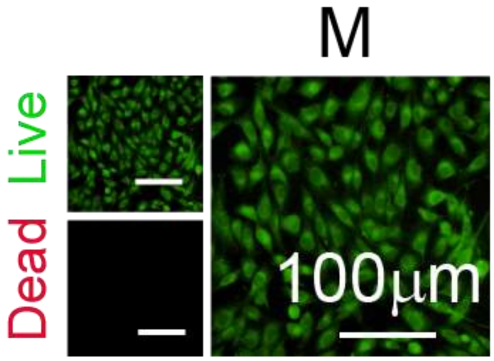

Figure S5. Live/dead staining of MG63 cells without culturing with the living material for 12 h in Transwell.

The "M" group represents that MG63 cells were seeded on the lower chamber of Transwell without hADSCs culturing on the upper chamber. As shown in Figure S5, almost no dead cells (stained red by PI) were observed, indicating the good cellular state of the tumor cells.

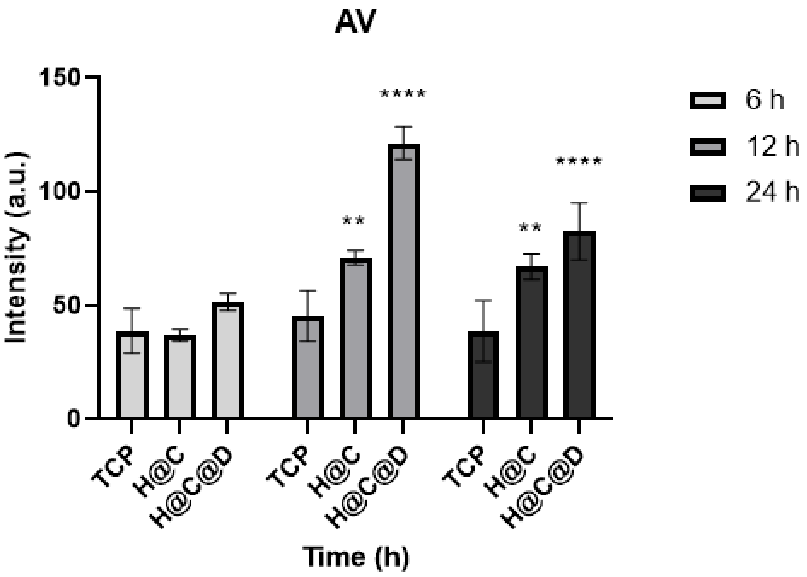

Figure S6. Statistical analysis of fluorescence intensity of AV-kFluor488 by AV/PI staining of MG63 cells after culturing with 100 μg/mL H@C and H@C@D for 6, 12 and 24 h. The p values were calculated using one-way ANOVA with Bonferroni's comparison test. (n = 3, *$p < 0.05$, **$p < 0.01$, and ***$p < 0.001$, ****$p < 0.0001$)

The fluorescence intensity of AV was increased 1.8-fold and 1.7-fold in H@C nanorods group for culturing 12 and 24 h and 3.1-fold and 2.1-fold in H@C@D nanorods group for culturing 12 and 24 h (Figure S6). The results indicated that the apoptosis was induced by the synergistic effect of H@C nanorods and Dox.

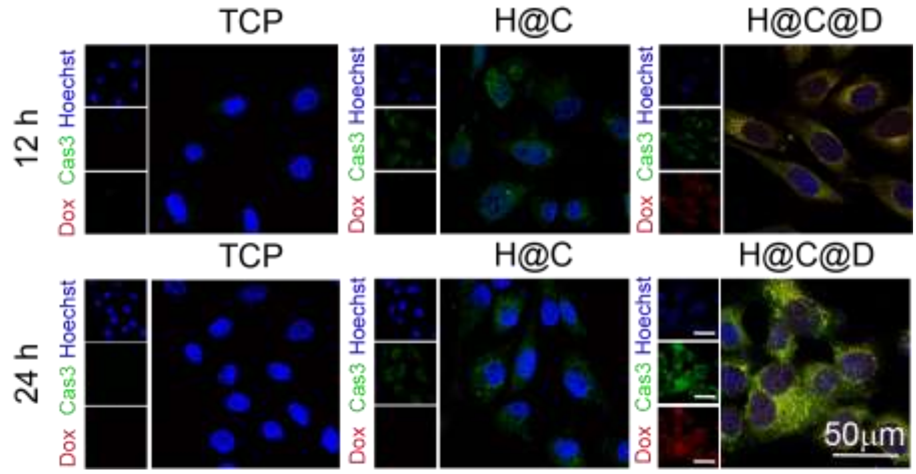

Figure S7. Caspase3 staining of MG63 cells. Caspase3 protein was stained green, Dox was red due to its autofluorescence and Nucleus was stained blue by Hoechst.

MG63 cells were cultured with 100 μg/mL H@C and H@C@D nanorods for 12 and 24 h. As shown in Figure S7, apoptosis related protein Caspase3 was expressed in MG63 cells after culturing with H@C nanorods for 12 and 24 h, indicating the anti-tumor ability of H@C nanorods. Furthermore, the expression level of apoptosis protein Cas3 and the amount of dox was the highest in MG63 culturing with H@C@D for 24 h. These results demonstrated that large amount of dox were released and accumulated in the cells for 24 h and promoted the apoptosis of MG63 cells. Additionally, the fluorescent intensity of Dox was increased after 24 h incubating compared to 12 h, demonstrating that Dox was successfully released from nanorods and play a role as an anti-tumor drug.

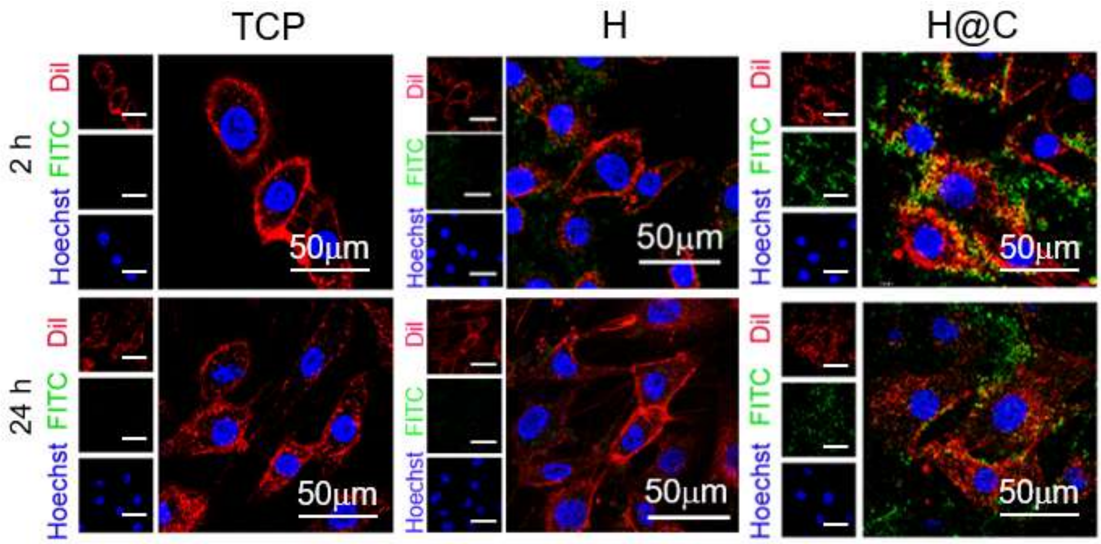

Figure S8. The colocalization of FITC-labeled nHAP, H@C and cell membrane of MG63 cells for 2 h and 24 h.

After culturing with nHAP for 2 and 24 h, the membrane of MG63 cells were stained red by Dil. As shown in Figure S8, the green luminescence which represents the nanorods was weak compared with H@C group which demonstrated that only a small amount of nanorods distribute around the MG63 cells for 2 h. And the green luminescence weakened after co-culturing for 24 h. Meanwhile, almost no yellow luminescence was observed, demonstrating that the colocalization of membrane and HAP nanorods was weak. However, H@C nanorods specifically linked to the membrane of MG63 cells after 2 h of culturing, accompanied by strong yellow luminescence. Interestingly, the yellow luminescence intensity was decreased with time prolonged. This revealed that some nanorods may fall off MG63 cells.

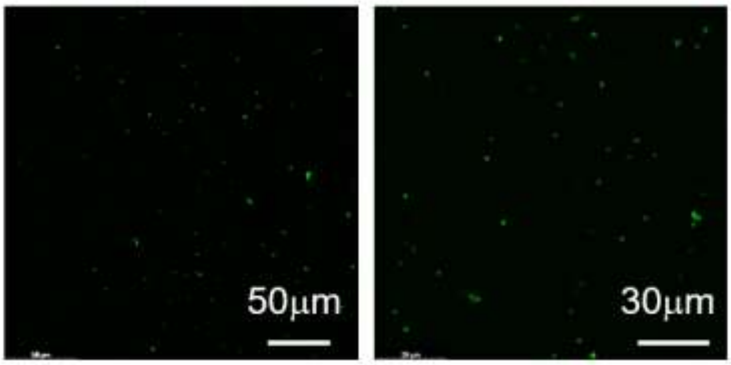

Figure S9. The deciduous H@C from the MG63 cells for 36 h.

To observe the detached H@C nanorods, MG63 cells were cultured with 100 μg/mL H@C nanorods in the Transwell insert for 2 h. Then, the basic medium was used to wash the ungrafted H@C from MG63. Subsequently, the insert was moved to the Transwell chamber and MEM medium was added in the lower chamber. After culturing for 36 h, the mixture in the lower chamber were collected and observed fluorescence inverted microscopy. As shown in Figure S9, some of the H@C nanorods will fall off the MG63 cells which may further play a role in modulated the fate of hADSCs.

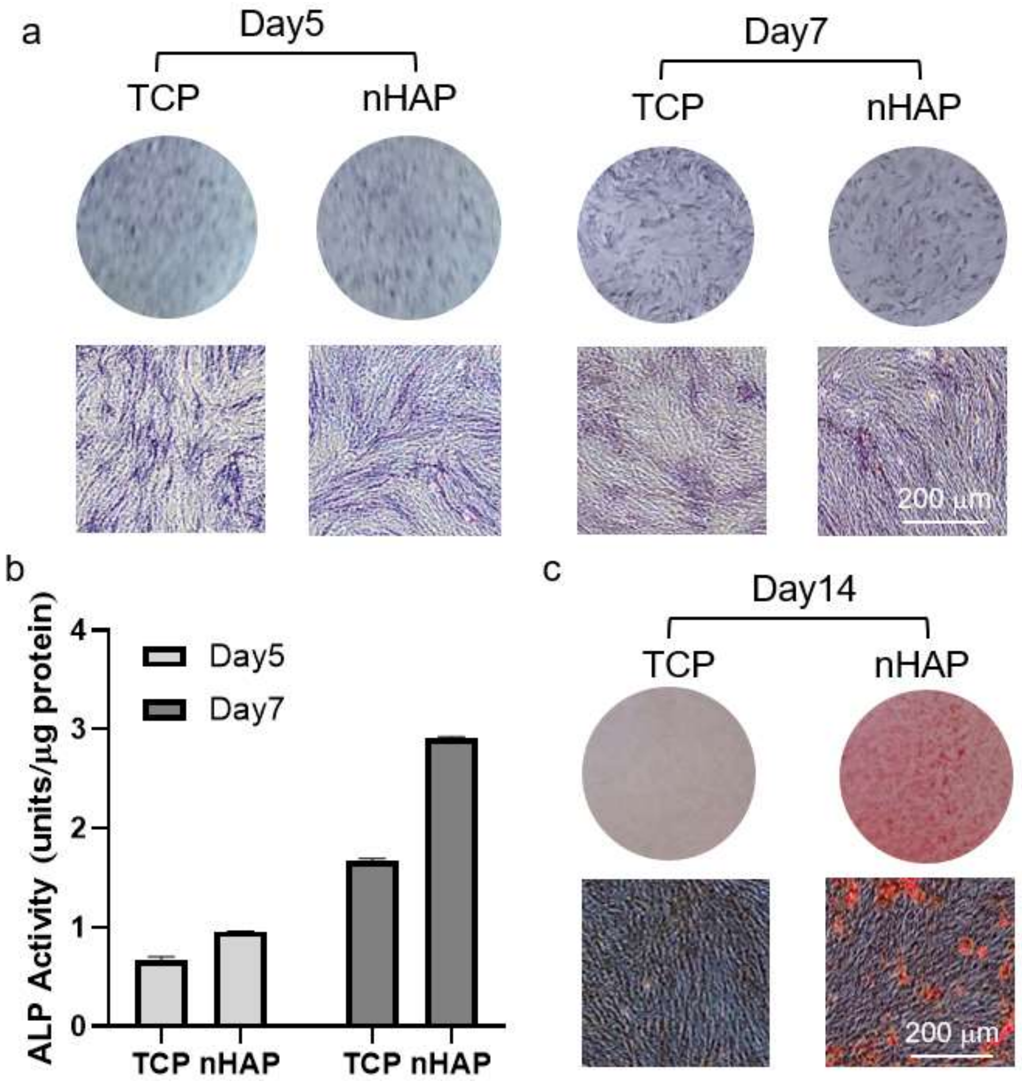

Figure S10. Osteogenic induction of hADSCs. (a, b) ALP staining and ALP activity of hADSCs after culturing with 100 μg/ml HAP nanorods for 5 and 7 days. (c) Alizarin red staining of hADSCs after culturing with100 μg/ml H@C nanorods for 14 days.

ALP is an early marker of osteogenic differentiation. As shown in Figure S10, the ALP activity is obviously improved in hADSCs cultured with 100 μg/ml HAP nanorods for 7 days, almost 1.7-fold of the control group. And the Alizarin red staining also demonstrated that nHAP nanorods could promote the osteogenic differentiation of hADSCs.

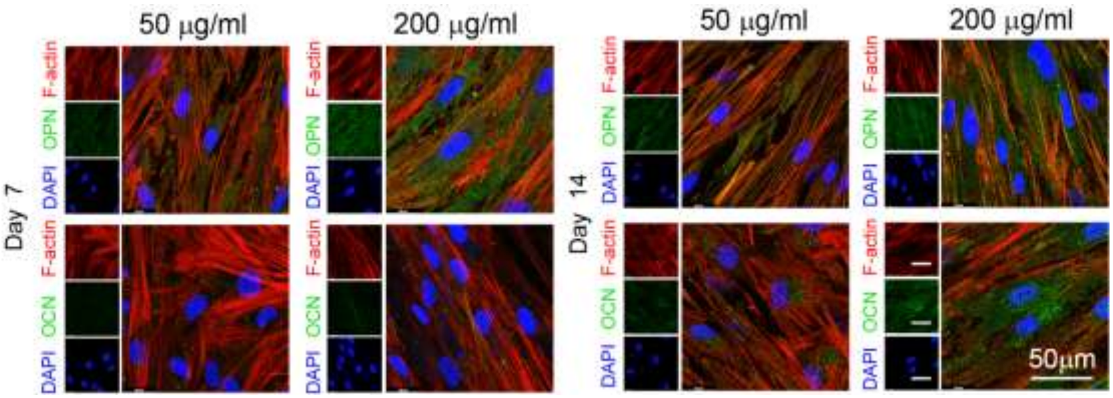

Figure S11. Immunofluorescence staining of osteogenic markers after culturing with 50 and 200 μg/ml H@C nanorods for 7 and 14 days.

The immunofluorescence staining of OPN and OCN in the hADSCs was observed by CLSM. After culturing with H@C nanorods for 7 and 14 days, F-actin, nuclei, OPN, and OCN protein of hADSCs were stained. As shown in Figure S11, the strong green luminescence which represent OPN protein was observed in 200 μg/ml H@C nanorods group at Day 7 and 14 which is obviously higher than control group. And, we can also observe the green luminescence which represent OCN protein in 50 and 200 μg/ml H@C group at day 7 while almost no green fluorescence was observed in control group. And OCN protein expressed more 200 μg/ml H@C nanorods group at day 14, indicating that 200 μg/ml H@C nanorods significantly improved the expression of OCN protein.

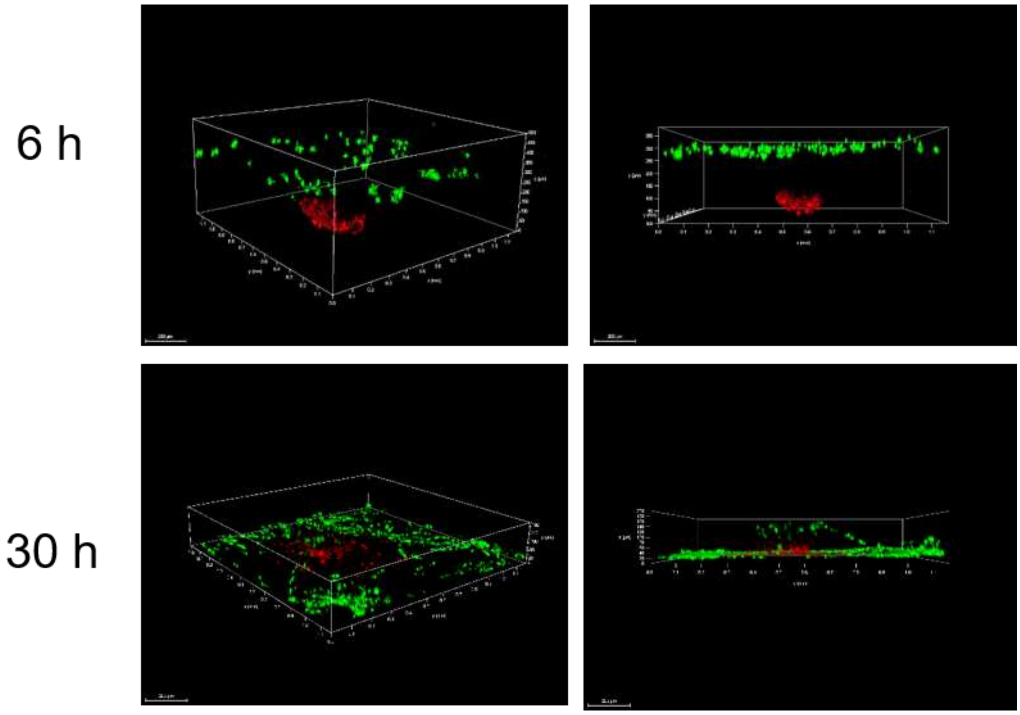

Figure S12. Top to Bottom 3D migration assay. H@C@D loaded hADSCs were labeled with pkh67 (Green) and MG63 spheres were labeled with pkh26 (Red).

As shown in Figure S12, the H@C@D loaded hADSCs were oberved on the top hydrogel layer at 6 h. After 30 h migration, the H@C@D loaded hADSCs were spread around tumor cells and tend to surround the tumor spheres. The results indicated the targeted migration ability of the living material.

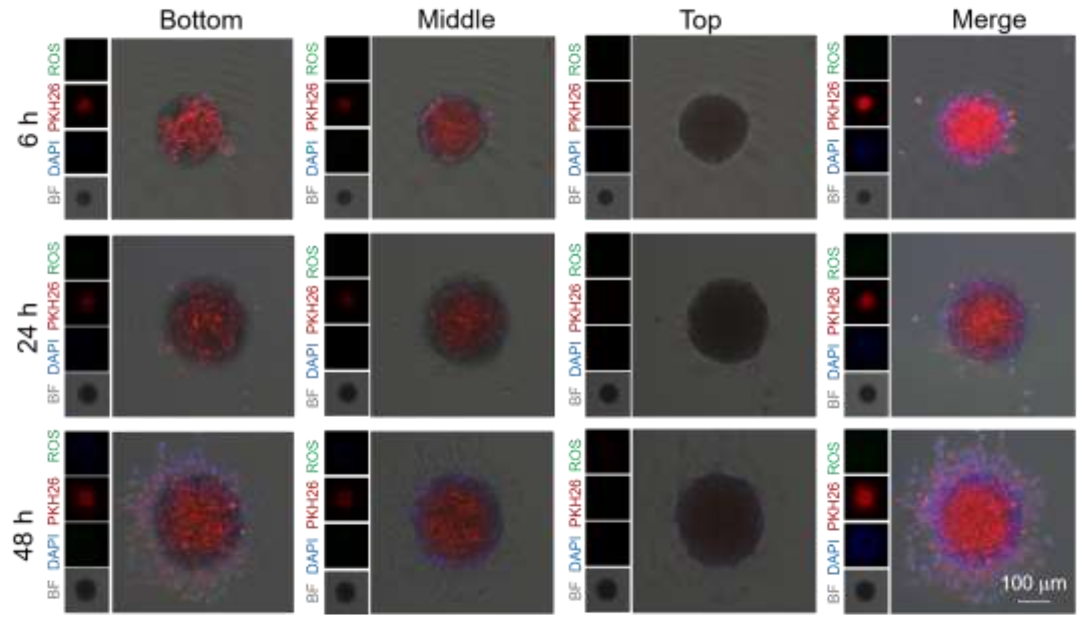

Figure S13. ROS staining of MG63 spheres in 3D microenvironment. Tumor spheres were labeled with pkh26 (Red), nucleus was stained blue by Hoechst and ROS was stained green. The different layer of MG63 spheres was observed by CLSM.

Ros staining of the negative control group was shown in Figure S13. Almost no ROS was detected in pure tumor spheres.

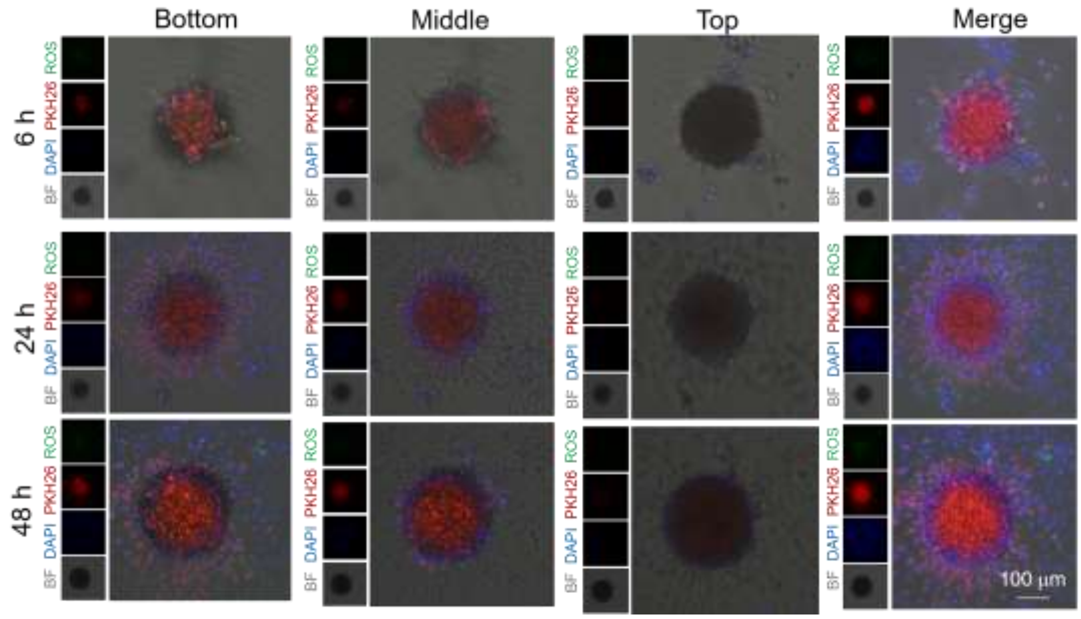

Figure S14. ROS staining of MG63 spheres and hADSCs in 3D microenvironment. Tumor spheres were labeled with pkh26 (Red), nucleus of hADSCs and tumor spheres were stained blue by Hoechst and ROS was stained green. The different layer of MG63 spheres was observed by CLSM.

As shown in Figure S14, fewer ROS was observed in the tumor spheres and hADSCs, indicating that pure hADSCs have little effect on kill the tumor cells.

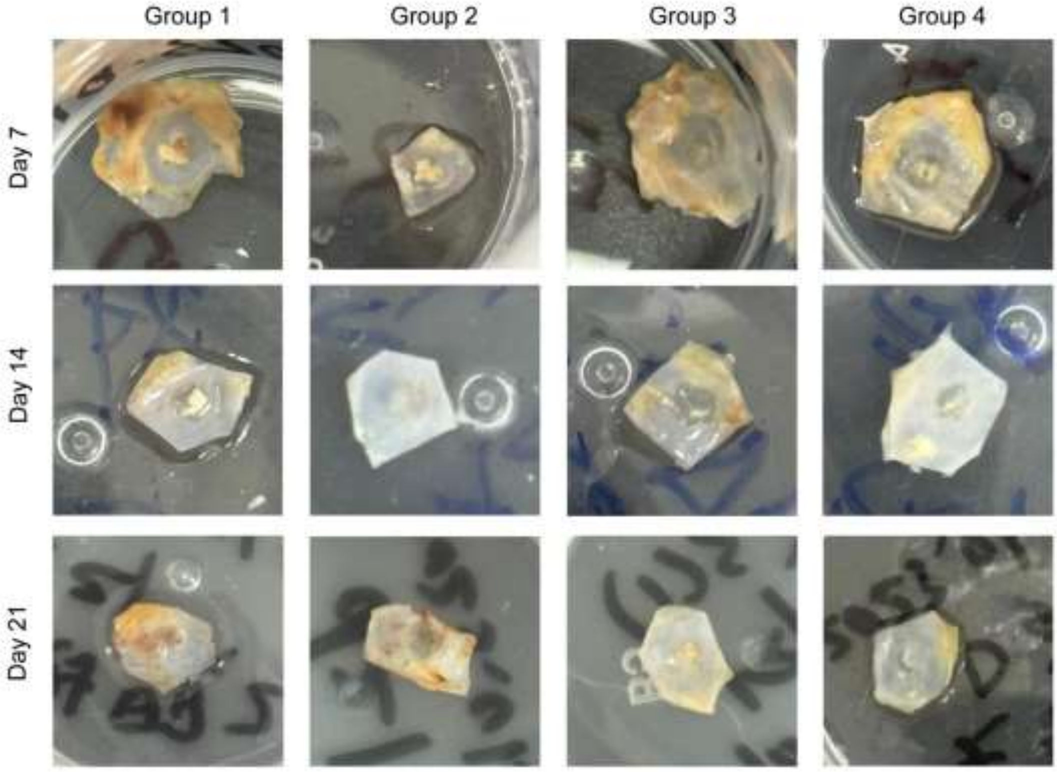

Figure S15. Photos of tissue samples from animal defect area.

Tissue samples were collected after 7, 14 and 21 days and then immersed in 4% paraformaldehyde solution. The defect area was clearly observed in the tissue samples. It showed that the defect was obviously reduced after 21 days culturing in Group 3 and Group 4.

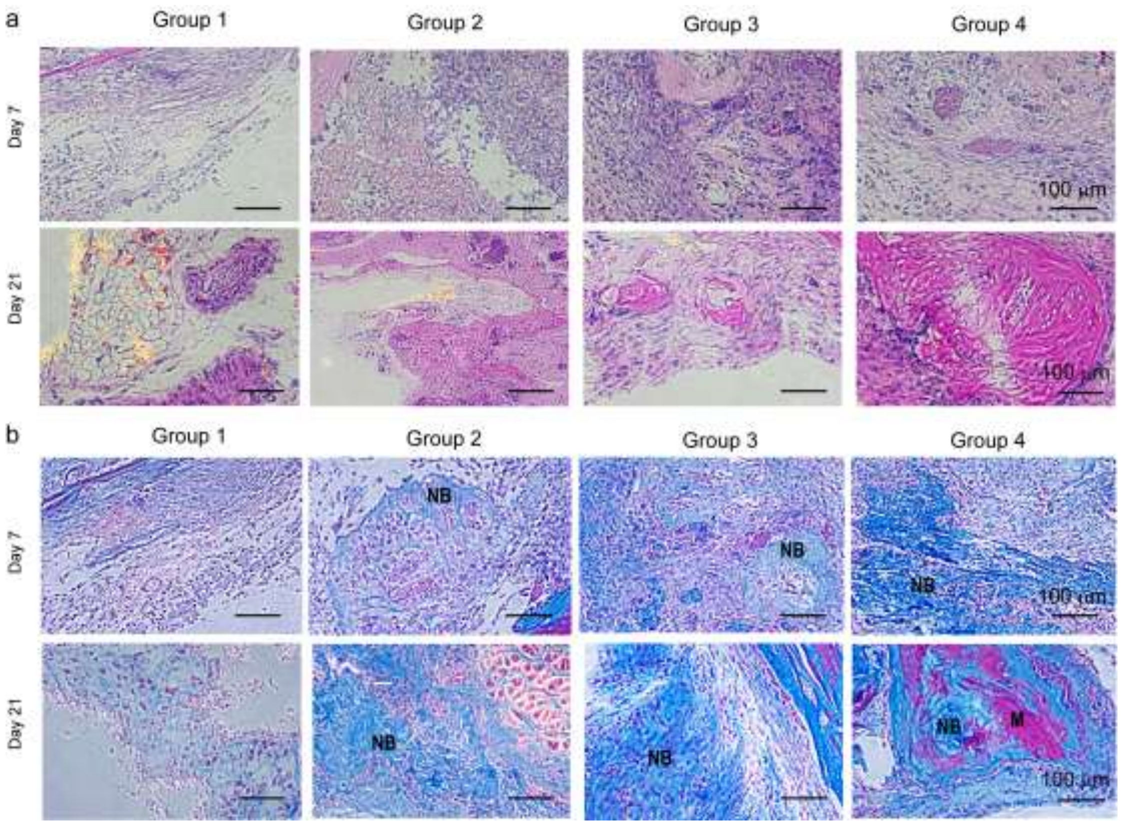

Figure S16. H&E staining and Masson-trichrome staining images of calvarial bone tissue sections at day 7 and 21.

After the bone defects were allowed to repair for 7 days, much more blue-stained collogen fiber was observed in the living material group (Group 4). We can also observe the newly formed bone in the H@C@D nanorods group. However, the defect area was still empty in control group (Group 1) and with low density of tissue and hADSCs group (Group 2). The results demonstrated the limited regenerative ability of hADSCs in the defect model with residual tumor cells. After 21 days repair, red-stained bone marrow was obviously observed in Group 4, indicating the good bone formation ability of the living material.

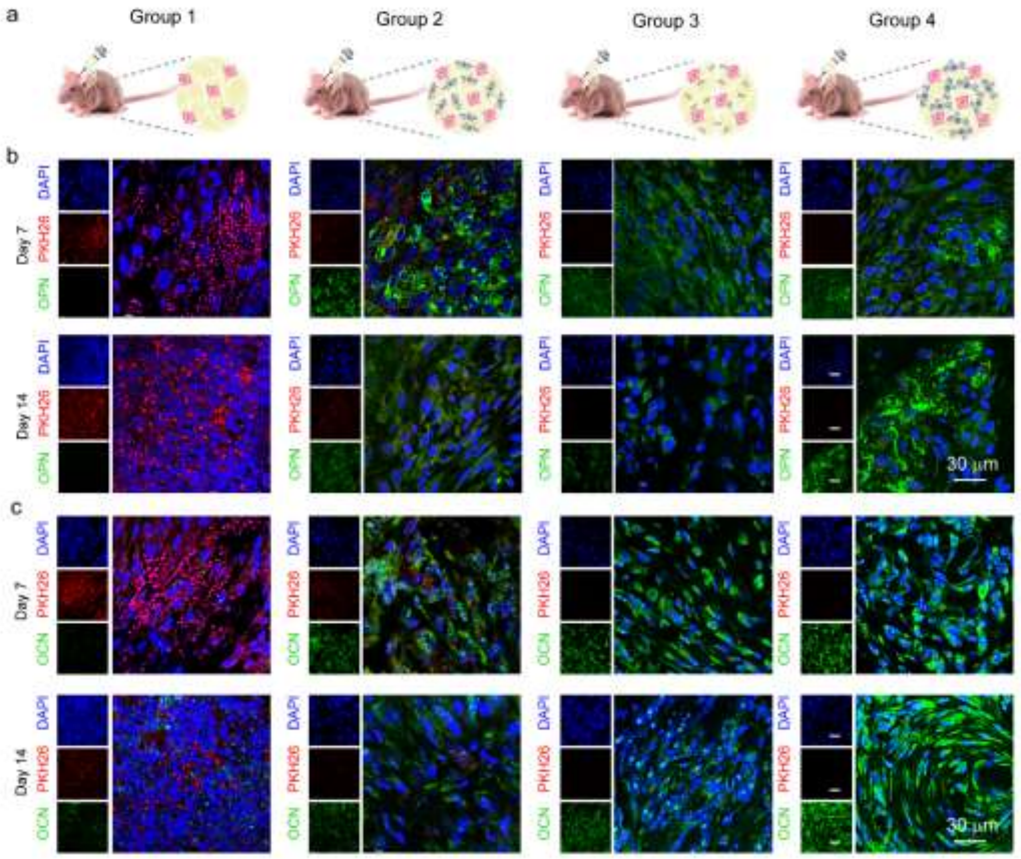

Figure S17. Immunostaining of tissue slices for the osteogenic markers and fluorescent images of PKH26-labeled tumor cells after 7 and 14 days. Cell nuclei were stained with DAPI (blue), OCN and OPN are shown in green, while tumor cells are in green. (a) The scheme of animal experiment. (b) OPN staining. (c) OCN staining.

After 7 days feeding, both three group have high level expression of OPN and OCN compare with control group (Group 1). However, obvious red-dye labeled tumor cells were observed in Group 2. The similar phenomenon was found in 14 days. It indicated that the limited anti-tumor ability of hADSCs, which would cause high risk for the recur of tumor cells. Also, the result indicated the good therapeutic effect of the H@C@D nanorods and the living materials.

Table S1. Sequences of RT-PCR primers.

Gene	Forward primers (5'-3')	Reverse primers (5'-3')
β-actin	CATGTACGTTGCTATCCAGGC	CTCCTTAATGTCACGCACGAT
OPN	CCTCCTAGGCATCACCTGTG	CCACACTATCACCTCGGCC
OCN	CTGTATCAATGGCTGGGAGC	GCCTGGAGAGGAGCAGAACT
RUNX2	AATGCCTCCGCTGTTATG	TTCTGTCTGTGCCTTCTTG
BMP2	ACCCTTTGTACGTGGACTTC	GTGGAGTTCAGATGATCAGC

Reference

[1] Zhang, P.; Zhang, Y.; Ding, X.; Shen, W.; Li, M.; Wagner, E.; Xiao, C.; Chen, *Adv Mater* **2020,** *32* (46), e2000013.
